# An Internet-Based Childhood Obesity Prevention Program (Time2bHealthy) for Parents of Preschool-Aged Children: Randomized Controlled Trial

**DOI:** 10.2196/11964

**Published:** 2019-02-08

**Authors:** Megan L Hammersley, Anthony D Okely, Marijka J Batterham, Rachel A Jones

**Affiliations:** 1 Early Start Faculty of Social Sciences University of Wollongong Wollongong Australia; 2 Statistical Consulting Service, National Institute for Applied Statistics Research Australia School of Mathematics and Applied Statistics University of Wollongong Wollongong Australia

**Keywords:** internet, eHealth, food intake, physical activity, screen time, sleep, self efficacy, body mass index

## Abstract

**Background:**

Electronic health (eHealth) obesity programs offer benefits to traditionally delivered programs and have shown promise in improving obesity-related behaviors in children.

**Objective:**

This study aimed to assess the efficacy of a parent-focused, internet-based healthy lifestyle program for preschool-aged children, who are overweight or at or above the fiftieth percentile for body mass index (BMI) for their age and sex, on child BMI, obesity-related behaviors, parent modeling, and parent self-efficacy.

**Methods:**

The *Time2bHealthy* randomized controlled trial was conducted in Australia, during 2016 to 2017. Participants were recruited both online and through more traditional means within the community. Parent or carer, and child (aged 2-5 years) dyads were randomized into an intervention or comparison group. Intervention participants received an 11-week internet-based healthy lifestyle program, underpinned by social cognitive theory, followed by fortnightly emails for 3 months thereafter. Intervention participants set goals and received individual feedback from a dietitian. They were also encouraged to access and contribute to a closed Facebook group to communicate with other participants and the dietitian. Comparison participants received email communication only. Objectively measured child BMI was the primary outcome. Secondary outcomes included objectively measured physical activity, parent-measured and objectively measured sleep habits, and parent-reported dietary intake, screen time, child feeding, parent modeling, and parent self-efficacy. All data were collected at face-to-face appointments at baseline, 3 months, and 6 months by blinded data collectors. Randomization was conducted using a computerized random number generator post baseline data collection.

**Results:**

A total of 86 dyads were recruited, with 42 randomized to the intervention group and 44 to the comparison group. Moreover, 78 dyads attended the 3- and 6-month follow-ups, with 7 lost to follow-up and 1 withdrawing. Mean child age was 3.46 years and 91% (78/86) were in the healthy weight range. Overall, 69% (29/42) of participants completed at least 5 of the 6 modules. Intention-to-treat analyses found no significant outcomes for change in BMI between groups. Compared with children in the comparison group, those in the intervention group showed a reduced frequency of discretionary food intake (estimate −1.36, 95% CI −2.27 to −0.45; *P*=.004), and parents showed improvement in child feeding pressure to eat practices (−0.30, 95% CI 0.06 to −0.00; *P*=.048) and nutrition self-efficacy (0.43, 95% CI 0.10 to 0.76; *P*=.01). No significant time by group interaction was found for other outcomes.

**Conclusions:**

The trial demonstrated that a parent-focused eHealth childhood obesity prevention program can provide support to improve dietary-related practices and self-efficacy but was not successful in reducing BMI. The target sample size was not achieved, which would have affected statistical power.

**Trial Registration:**

Australian New Zealand Clinical Trials Registry ANZCTR12616000119493; https://www.anzctr.org.au/ Trial/Registration/TrialReview.aspx?id=370030 (Archived by WebCite at http://www.webcitation.org/74Se4S7ZZ).

## Introduction

### Background

The World Health Organization (WHO) has described childhood obesity as one of the most significant public health issues [1]. Around 23% of children and adolescents in developed countries and 13% in developing countries are overweight or obese [2]. One of the main influences on the development of childhood obesity is parental guidance and role modeling around obesity-related behaviors [3-5], particularly in the early years of life up to 5 years of age [5]. Health behaviors become more difficult to change with age [5] and tend to track into adulthood [6], but are quite malleable in the early years [5]. Therefore, early childhood is an opportune time to intervene, and involving parents in interventions appears to be crucial [7].

Targeted interventions have the potential to alter the trajectory of childhood overweight and obesity continuing into adulthood, and interventions that involve parents are the most successful [8-10]. However, barriers to traditional face-to-face interventions such as scheduling of appointments [10], stigma, parental denial [11], childcare for other siblings [12], travel [13], and cost [10] can prevent sustained parental involvement and commitment and, therefore, potentially impact the success of interventions.

Overweight and obesity interventions, which use an electronic health (eHealth) delivery method, offer many advantages compared with traditional delivery methods, particularly around convenience and accessibility. Most interventions using eHealth delivery methods have been conducted in older children and have not involved parents [14]. In a recent meta-analysis of parent-focused eHealth obesity interventions for 0- to 18-year-olds, around half of the included studies showed significant improvements in the dietary intake or physical activity when compared with a control group, but there was no significant change in the body mass index (BMI)/BMI z-score. In this review, no studies targeting children aged under 5 years were included, and it was recommended that larger, higher-quality parent-focused eHealth studies be conducted, with a particular focus on younger age groups [14]. There is also a lack of studies that focus on obesity-related behaviors beyond dietary intake and physical activity. It is important that interventions focus on total movement throughout the day and incorporate strategies to improve sleep and reduce sedentary behavior, aligning with the recommendations of newly released 24-hour movement guidelines [15,16]. Furthermore, although some studies have been underpinned by social cognitive theory [17-21], few have assessed change in parent self-efficacy, a key construct of social cognitive theory.

### Objectives

This paper reports the outcomes of a randomized controlled trial (RCT) evaluating the efficacy of a parent-focused, internet-based program in facilitating behavior change in preschool-aged children who are overweight or at risk of becoming overweight. We hypothesized that children in the intervention group would achieve significantly greater reductions in BMI compared with those in the comparison group at 6-month follow-up. It was also hypothesized that the intervention group would achieve significantly greater improvements in child dietary intake, physical activity, screen time, sleep, child feeding, and parent self-efficacy and role modeling.

## Methods

### Study Design

The protocol for this study has been published [22]. Briefly, the *Time2bHealthy* study was based on formative research with parents of preschool-aged children [23] and was piloted [24] before this trial. This study was a 2-arm parallel RCT involving parent-child dyads recruited into 6 cohorts. The trial was conducted between January 2016 and December 2017 in the Illawarra, Southern and South-Western Sydney, Southern Highlands, and Shoalhaven areas of New South Wales and Melbourne, Victoria, Australia. Measures were collected at baseline, 3 months post baseline, and 6 months post baseline. The primary outcome was change in BMI 6-months post baseline. The 6-month time point was selected as it was not expected that the 3-month time point would provide adequate time to detect changes in BMI. Secondary outcomes included child dietary intake, physical activity, screen time, sleep, child-feeding practices, and parent self-efficacy and role modeling.

The Consolidated Standards of Reporting Trials statement was used to guide the reporting of this study [25]. The study was registered with the Australian and New Zealand Clinical Trials Registry (12616000119493) and approved by the University of Wollongong Human Research Ethics Committee (HE15/354).

### Participant Recruitment and Eligibility Criteria

Potential participants were informed about the study through flyers distributed at early childhood education and care centers, general practices/primary health care centers, early childhood health centers, playgroups, and local sporting groups. Flyers were also displayed on community notice boards (eg, libraries, shopping centers, children’s activity centers), and articles were placed in the University of Wollongong and Local Health District newsletters and posted on Facebook. Media releases were also sent to local media outlets.

As the focus of the program was prevention of childhood obesity, healthy weight children as well as overweight children were included in the sample. Participants were eligible if they had access to the internet, if their child was 2 to 5 years old (and not yet attending school), and was at or above the WHO fiftieth percentile for BMI for their age and sex [26,27], a criterion used in other similar studies [17-19]. Parents also needed to have a Facebook account or agreed to create one.

Child participants were excluded if they were taking medications or had a medical condition with the potential to affect weight or restrict age-appropriate play. Children with conditions that required the restriction of certain foods (eg, celiac disease or food allergies) were deemed eligible to participate, but parents were informed that parts of the program would not be completely appropriate and that they would need to make some adaptations to the material provided to match their child’s individual dietary/health needs.

Informed written consent was provided by the parents/guardians after reading a participant information sheet. Provisional eligibility was determined through contact with participants via phone or email and was confirmed at the face-to-face baseline data collection visit when the child’s height and weight were measured to confirm if the child’s BMI was at or above the WHO fiftieth percentile for age and sex. Participants below the fiftieth percentile were excluded.

### Randomization and Blinding

Participants were randomized into the intervention or comparison group following the collection of baseline measures. Randomization was performed in a 1:1 ratio using a computerized random number generator. A data manager with no other involvement in the study conducted the randomization. The researcher responsible for implementing the intervention was the only person who was informed about group allocation. At the follow-up data collection time points, height and weight measurements were taken by trained data collectors blinded to group allocation.

### Time2bHealthy Intervention

Participants randomized to the intervention group were provided with an individual log-in to access the *Time2bHealthy* program. The development, content, and theoretical framework for this intervention have been previously published [22]. Briefly, the intervention was guided by Bandura’s social cognitive theory [28] and was designed using a backwards intervention mapping process [29,30]. The intervention targeted multiple behaviors and consisted of 6 modules including an introduction, nutrition (n=2), physical activity, screen time, and sleep module, which were completed by the participants over an 11-week period. Each module comprised reading material, videos, activities, quizzes, and a goal-setting component. Participants received feedback on their goals at the end of each module by a dietitian and were provided with advice to improve their goals using the SMART (Specific, Measurable, Attainable, Realistic, Timely) goal framework [31]. Participants also received weekly emails reminding them to log on to the website and participate in the activities. Participants were informed that they could make contact via email or phone if they had questions or concerns at any time. Participants in each of the cohorts were also encouraged to access and contribute to a closed (secret) Facebook group to communicate with other members of the cohort and the dietitian. There was a separate group for each cohort, and they were regularly monitored and moderated by the dietitian. Participants were asked to post photos, recipes, and personal experiences and ideas that they had found helpful for behavior change, which were relevant to each module. If the dietitian could not answer a question raised, advice was sought from another member of the research team, which included experts in physical activity. An incentive to post to the group was provided, with 1 post being selected from each module (2 to 6) to receive a gift card.

Participants continued to receive emails fortnightly at the end of the program until the 6-month follow-up. Infographics summarizing the key points from each of the modules were provided in these emails, and participants were also encouraged to log back into the website to revise the material and review their progress with their goals.

### Comparison Condition

Participants randomized to the comparison group received fortnightly emails, which contained links to the *Raising Children Network* website (an Australian government-funded parenting website). The topics were similar to *Time2bHealthy* (nutrition, physical activity, screen time, and sleep) and also included other general health information. There were no interactive components available to this group. After the final data collection point at 6 months, participants from this group were provided access to *Time2bHealthy*, but they did not receive access to a Facebook group or to the regular emails.

### Outcome Measures

Measurements were taken at baseline and 3 and 6 months post baseline. Participant measures were collected at the University of Wollongong, in the participant’s home, or in a community setting. Questionnaires were completed by the parents on an iPad during these sessions, which took approximately 30 to 45 min. Demographic information was also collected from parents at the baseline data collection point. Participants in the intervention group were asked to complete a process evaluation questionnaire at the end of the Web-based program, which assessed user acceptability of the program content, length, goal setting, Facebook discussion group, and the modality used.

#### Primary Outcome Measure

Child height and weight were measured using a standardized method [32] to calculate BMI. A stadiometer was used to measure height to the nearest 0.1 mm. Weight was measured (with no shoes and minimal clothing) to the nearest 0.1 kg using a Seca scale. Both height and weight were measured twice. The mean of these 2 measurements was used to calculate BMI. A third measurement was taken when height measurements differed by more than 0.5 cm and weight measurements differed by more than 0.5 kg.

#### Secondary Outcome Measures

Dietary intake was assessed using both a parent-reported food questionnaire (modified from the Eating and Physical Activity Questionnaire) [33] and a parent-reported 24-hour recall of child dietary intake (using the “Easy Diet Diary” app [Xyris Software, Australia, Pty Ltd]). The section of the food questionnaire, which asked about the frequency of intake of discretionary foods, was expanded to include additional discretionary food categories, which used the same scale as the existing question. Cronbach alpha=.68 for these discretionary food questions. Data from the 24-hour recall was used to calculate kJ per kg of body weight, percentage of kJ from sugar, and percentage of kJ from saturated fat. Data from the food questionnaire were used to assess the daily fruit intake, daily vegetable intake, and frequency of fruit juice and sugary drinks intake. A discretionary food score was calculated based on responses to questions on the frequency of intake of takeaway or fast food; sugary cereals; potato chips or other salty snacks; sweets; cakes, doughnuts, and sweet cookies, or muffins.

Physical activity intensity and duration were measured using an ActiGraph GT3X+ accelerometer (ActiGraph Corporation, Pensacola, FL), which was worn on an elasticized belt around the child’s waist for 7 days. Accelerometer data were analyzed in ActiLife version 6 (ActiGraph Corporation, Pensacola, FL). A sampling frequency of 30 Hz was used, with the files then reintegrated into 15-second epochs. Nonwear time was defined as 20 min or more of 0 counts. Accelerometer data used for the physical activity analysis were considered valid based on wear time of at least 6 hours per day on 3 days, which has been found to be reliable in previous research [34]. The following cut points appropriate for preschool-aged children were used to categorize physical activity intensity: sedentary, <100 counts/min; low light–intensity physical activity, 101 to 800 counts/min; high light–intensity physical activity, 801 to 1679 counts/min; moderate-intensity physical activity, 1680 to 3367 count/min; and vigorous-intensity physical activity, ≥3368 count/min [35].

Sleep habits were assessed using 4 questions assessing sleep latency, sleep reluctance, difficulty sleeping, and difficulty falling to sleep in own bed based on questions from the Children’s Sleep Habits Questionnaire [36] (Cronbach alpha=.63 for the 3 scaled questions relating to sleep reluctance, difficulty falling asleep, and difficulty falling to sleep in own bed) and questions about the child’s usual sleep and wake times and an Actigraph GT3X+ accelerometer. Sleep accelerometer data were analyzed in ActiLife using the Sadeh algorithm, which is appropriate for use in children [37]. Sleep accelerometer data were considered valid based on a wear time of at least 3 nights [38].

Parent-reported questionnaires were used to assess child feeding (from the Child Feeding Questionnaire predefined subscales of “restriction” and “pressure to eat” [39]), screen time (based on the studies by Downing et al and Hinkley et al [40,41] and additional questions relating to screen entertainment rules, presence of a television in the child’s bedroom and frequency of watching television while eating a meal), parent modeling (developed after reviewing the studies by Palfreyman et al and Gattshall et al [42,43]; Cronbach alpha=.63), and parent self-efficacy in nutrition, physical activity, screen time, and sleep (modified from Bohman et al [44] by adding 6 additional questions and making small changes to some existing questions to align the questionnaire to the program content; Cronbach alpha=.89).

### Power and Sample Size

On the basis of the results of the pilot study [24], we expected a BMI effect size of approximately 0.4 for this trial. To detect a statistically significant difference between groups (alpha=.05 and power=.8), 136 participants were required (68 per group), and based on an estimated attrition rate of 15%, we aimed to recruit 160 participants (80 per group).

### Statistical Analyses

Differences in changes over time between the intervention and comparison groups were assessed for each outcome. Linear mixed models were used to determine differences between groups over time (baseline, 3 months, and 6 months) with adjustment for potential covariates. Intention-to-treat (ITT) principles were used for parametric data, with all participants analyzed in the group to which they were randomized regardless of whether they attended all data collection time points or completed the intervention. Covariates included baseline values, age, and cohort. Due to nonparametric distributions for some variables, Freidman tests and Wilcoxon signed rank tests were used followed by Mann-Whitney tests to analyze nonparametric data using completed cases. Generalized estimating equations were considered; however, the analyses would not converge.

Post hoc analysis of covariance (ANCOVA) analyses were used to detect changes between groups at individual time points, which included the baseline value, age, and cohort as covariates. Within-group changes were analyzed using repeated measures analysis of variance (ANOVA), which included age and cohort as covariates. These were complete case analyses. Analyses were performed using IBM SPSS Statistics for Windows, version 25 (IBM Corp, Armonk, NY, USA).

## Results

### Overview

**Figure 1:**
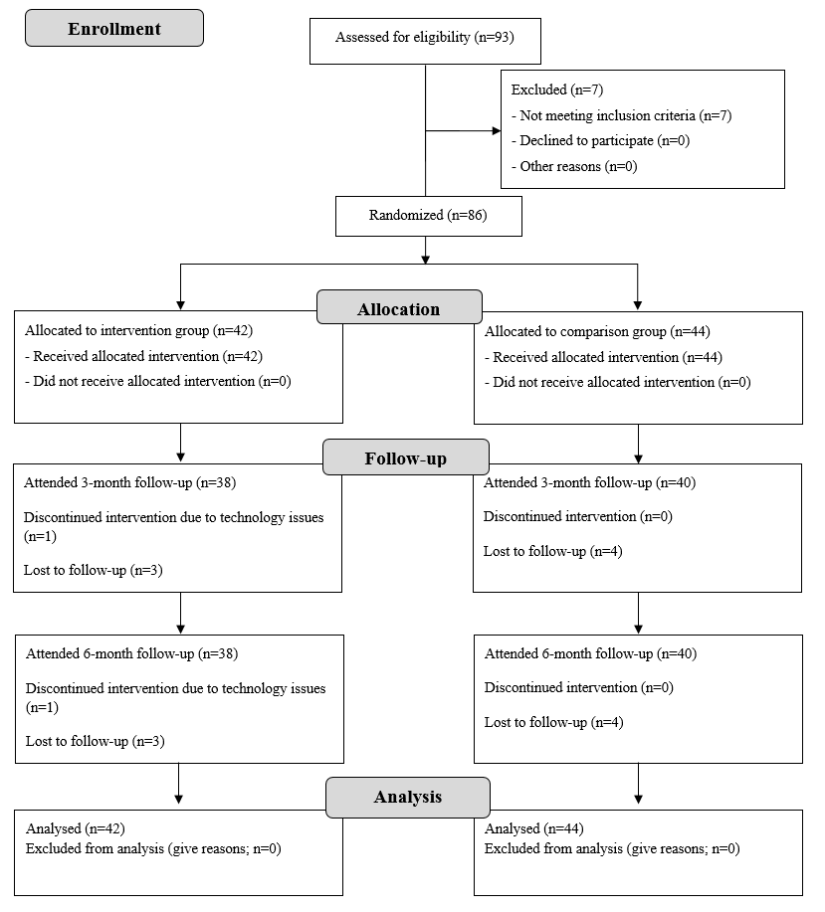
CONSORT flow diagram for *Time2bHealthy* randomized controlled trial.

**Figure 2:**
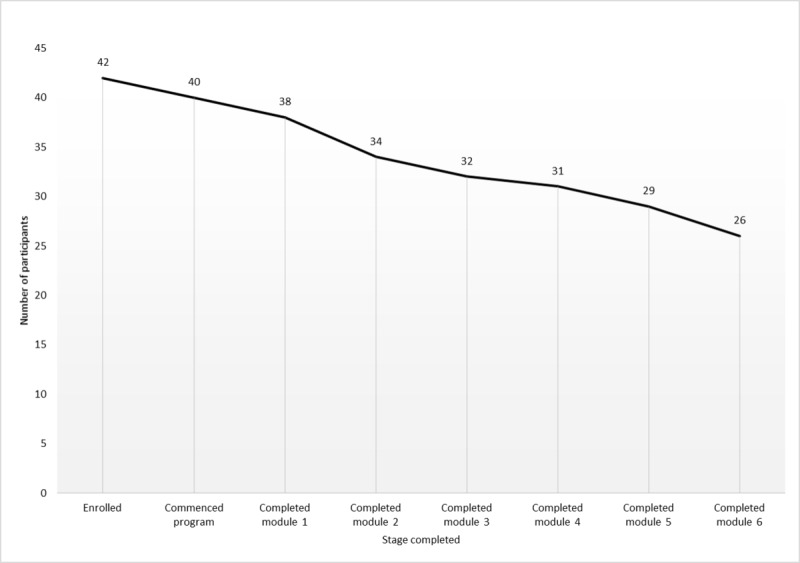
Participant completion of *Time2bHealthy* modules.

Figure 1 shows the flow of participants through the study. Recruitment was conducted between January 2016 and June 2017. Enquiries were received from 372 parents initially. After viewing the information sheet, 159 parents remained interested in the study and were screened via phone or email, with 104 being potentially eligible. Of the 93 parent-child dyads who attended the initial visit, 86 were eligible and enrolled in the study. A total of 42 participant dyads were randomized to the intervention group and 44 to the comparison group. The mean number of participants per cohort was 14 (range 8-22), and the mean number of participants in each Facebook group was 6 (range 3-10). Follow-up was conducted between July 2016 and December 2017. Moreover, 78 participants (91%) attended the 3- and 6-month follow-ups, with 7 (8%) lost to follow-up and 1 participant (1%) withdrawing from the intervention group due to problems accessing the internet. Figure 2 shows the completion of each of the intervention program modules. At least 5 of the 6 modules were completed by 29 participants (69%).

### Participant Characteristics

The baseline characteristics of participants are displayed in Table 1. The mean age (SD) of the participating children was 3.46 (0.92) years, and 50% of child participants were female. The mean age (SD) of the participating parents was 35.17 (4.80) years and 97% (83/86) were female, 63% (54/86) had a university degree, 50% (43/86) had an after-tax income of at least Aus $580/week, and 85% (73/86) were married or had a partner. The majority of children were in the healthy weight range (78/86, 91%) according to the WHO criteria [1]. The mean (SD) BMI of the participating children was 17.01 (1.24). The mean (SD) BMI of participating parents was 26.08 (5.97), and 45% (39/86) were overweight or obese.

### Primary Outcome

Table 2 displays the baseline, 3-month, and 6-month BMI results. The results of the ITT, displayed in Table 3, indicated that there was no group-by-time effect for BMI. The ANCOVA analyses (shown in Tables 4 and 5) also found no significant differences between groups at each time point. When considering changes within groups, the repeated-measures ANOVA found a significant change in BMI within the intervention group at both the 3-month (adjusted mean difference −.26, 95% CI −0.51 to −0.02; *P*=.03) and 6-month time points (adjusted mean difference −0.22, 95% CI −0.40 to −0.03; *P*=.02) and no significant changes within the comparison group.

### Secondary Outcomes

Table 2 displays the baseline, 3-month, and 6-month results for parametric secondary outcomes. The linear mixed model analyses (displayed in Table 3) found a significant group-by-time interaction for frequency of consumption of discretionary foods (estimate −1.36, 95% CI −2.27 to −0.45; *P* ≤.01), nutrition parent self-efficacy (estimate 0.43, 95% CI 0.10 to 0.76, *P*=.01), and child feeding–pressure to eat (estimate −0.30, 95% CI 0.61 to −0.00, *P*=.048). No group-by-time interaction effects for any other secondary outcomes were observed.

The posthoc ANCOVA analyses results (displayed in Table 4 and 5) showed a significant difference between groups in frequency of consumption of discretionary foods at 3 months (adjusted mean difference −1.45, 95% CI −2.42 to −0.43; *P*=.01) and 6 months (adjusted mean difference −1.30, 95% CI −2.34 to −0.26; *P*=.02), nutrition parent self-efficacy at 6 months (adjusted mean difference 0.53, 95% CI 0.13 to 0.93; *P*=.01), child feeding–pressure to eat at 6 months (adjusted mean difference −0.35, 95% CI −0.68 to −0.02; *P*=.04), and accelerometer-measured sleep duration (in the nonhypothesized direction) at 6 months (adjusted mean difference −0.55, 95% CI −1.01 to −0.03; *P*=.04). The results of the Mann-Whitney tests for the nonparametric data showed that there were no significant differences between groups for any parameter (at Bonferroni adjusted *P*<.008).

**Table 1:** Baseline characteristics of participants.

Variable		Comparison group (n=44)	Intervention group (n=42)	All (n=86)
**Child (sex), n (%)**				
	Male	19 (43)	24 (57)	43 (50)
	Female	25 (57)	18 (43)	43 (50)
Child age (months), mean (SD)		43 (12.26)	40 (9.65)	42 (11.05)
Child age (years), mean (SD)		3.55 (1.02)	3.36 (0.80)	3.46 (0.92)
Child body mass index (BMI), mean (SD)		16.72 (0.92)	17.28 (1.44)	17.01 (1.24)
**Child weight status^a^, n (%)**				
	Healthy weight	38 (86)	40 (95)	78 (91)
	Overweight	5 (11)	2 (5)	7 (8)
	Obese	1 (2)	0 (0)	1 (1)
Median BMI percentile range		85 to ≤95	75 to ≤85	75 to ≤85
**Child aboriginal/Torres Strait Islander status, n (%)**				
	Aboriginal	4 (9)	1 (2)	5 (6)
	Torres Strait Islander	0 (0)	0 (0)	0 (0)
	No	39 (89)	40 (95)	79 (92)
	Not answered	1 (2)	1 (2)	2 (2)
**Participating parent’s sex, n (%)**				
	Male	1 (2)	2 (5)	3 (3)
	Female	43 (98)	40 (95)	83 (97)
Participating parent’s age, mean (SD)		34.91 (4.68)	35.45 (4.95)	35.17 (4.80)
**Highest level of education of the participating parent, n (%)**				
	Not university qualified	22 (50)	8 (19)	30 (35)
	University qualified	22 (50)	32 (76)	54 (63)
	Currently studying	0 (0)	2 (5)	2 (2)
**Participating parent’s income after tax (Aus $), n (%)**				
	<$580/week	23 (52)	20 (48)	43 (50)
	$580-$1240/week	15 (34)	16 (38)	31 (36)
	>$1240/week	6 (14)	6 (14)	12 (14)
BMI of participating parent, mean (SD)		27.38 (21.61)	24.81 (4.64)	26.08 (5.97)
**Weight status of participating parent, n (%)**				
	Underweight	1 (2)	1 (2)	2 (2)
	Healthy weight	15 (34)	26 (62)	42 (49)
	Overweight	13 (30)	9 (21)	22 (26)
	Obese	11 (25)	6 (14)	17 (20)
	Not answered	3 (7)	0 (0)	3 (3)
**Aboriginal status of participating parent, n (%)**				
	Aboriginal	2 (4.55)	1 (2.38)	3 (3.49)
	No	41 (88.64)	40 (95.24)	81 (94.19)
	Not answered	1 (2.27)	1 (2.38)	2 (2.33)
**Participating parent’s relationship with child, n (%)**				
	Biological mother	41 (93)	39 (93)	80 (93)
	Biological father	2 (5)	2 (5)	4 (5)
	Other	1 (2)	1 (2)	2 (2)
**Marital status of participating parent, n (%)**				
	Single/divorced/separated/widowed	10 (23)	3 (7)	13 (15)
	Married/with partner	34 (77)	39 (93)	73 (85)
BMI of other parent, mean (SD)		27.61 (4.51)	28.24 (6.72)	27.95 (5.76)
**Weight status of other parent, n (%)**				
	Underweight	0 (0)	0 (0)	0 (0)
	Healthy weight	9 (20)	15 (36)	24 (28)
	Overweight	13 (30)	9 (21)	22 (26)
	Obese	9 (20)	11 (26)	20 (23)
	No answer/not applicable	13 (30)	7 (17)	19 (22)
**Income of other parent (Aus $), n (%)**				
	<$580/week	6 (14)	5 (12)	11 (13)
	$580-$1240/week	20 (45)	19 (45)	39 (45)
	>$1240/week	9 (20)	15 (36)	24 (28)
	No answer/not applicable	9 (20)	3 (7)	12 (14)
**Language spoken at home, n (%)**				
	English	40 (91)	37 (88)	77 (90)
	Other	4 (9)	5 (12)	9 (10)
**Found out about the program, n (%)**				
	Early childhood education center	18 (41)	16 (38)	34 (40)
	Flyer	5 (11)	7 (17)	12 (14)
	Early childhood nurse/center	2 (5)	5 (12)	7 (8)
	Email	0 (0)	4 (10)	4 (5)
	School newsletter	2 (5)	1 (2)	3 (3)
	Media (print, television, and radio)	2 (5)	1 (2)	3 (3)
	Social media	5 (11)	4 (10)	9 (10)
	Playgroup	3 (7)	0 (0)	3 (3)
	Other	7 (16)	4 (10)	11 (13)

^a^World Health Organization definition [1].

**Table 2:** Mean (SD) values for primary and secondary outcomes at each time point.

Variable		Baseline, mean (SD)		3 months, mean (SD)		6 months, mean (SD)	
		Comparison (n=44)	Intervention (n=42)	Comparison (n=40)	Intervention (n=38)	Comparison (n=40)	Intervention (n=38)
Body mass index (BMI)		17.28 (1.44)	16.72 (0.92)	16.99 (1.25)	16.46 (0.80)	16.87 (1.24)	16.51 (0.75)
Median BMI percentile range		85 to ≤95	75 to ≤85	85 to ≤95	75 to ≤85	85 to ≤95	75 to ≤85
kJ/kg of body weight^a^		330.43 (125.08)	343.64 (112.01)	296.24 (114.64)	303.75 (120.15)	296.20 (82.05)	327.60 (104.06)^b^
Percentage of kJ from sugar^a^		22.24 (6.75	20.14 (7.01)	21.15 (7.30)	20.83 (6.02)	19.29 (7.01)	19.54 (6.95)^b^
Percentage of kJ from saturated fat^a^		12.52 (4.77)	11.74 (3.95)	11.58 (3.84)	11.37 (3.91)	12.50 (3.74)	11.00 (3.90)^b^
Servings of fruit^c^		2.91 (1.03)	2.52 (0.92)	2.95 (0.96)	2.47 (0.83)	2.88 (1.04)	2.53 (0.86)
Servings of vegetables^c^		2.34 (1.08)	2.62 (1.27)	2.53 (1.22)	2.84 (1.22)	2.65 (1.05)	2.97 (1.28)
Discretionary food frequency score^d^		11.73 (2.86)	11.21 (3.82)	11.60 (2.73)	9.82 (3.21)	11.90 (2.29)	10.40 (3.22)
Nutrition self-efficacy^e^		7.94 (1.13)	8.19 (1.36)	8.28 (1.19)	8.69 (0.97)	8.30 (1.22)	8.89 (0.89)
Child feeding–restriction^f^		3.60 (0.92)	3.630 (0.78)	3.73 (0.84)	3.69 (0.75)	3.58 (0.89)	3.66 (0.79)
Child feeding–pressure^f^		2.34 (0.98)	2.52 (0.99)	2.34 (1.09)	2.17 (1.08)	2.43 (1.04)	2.14 (0.99)
Parent modeling^g^		3.95 (0.76)	3.98 (0.79)	3.93 (0.85)	4.18 (0.55)	4.16 (0.73)	4.36 (0.54)
Sleep reluctance^h^		3.00 (1.24)	2.36 (1.06)	2.65 (1.00)	2.13 (0.99)	2.68 (0.97)	2.24 (1.14)^b^
**Sleep**		**n=34**	**n=34**	**n=19**	**n=28**	**n=20**	**n=21**
	Duration (hours)^i^	9.59 (0.93)	9.85 (0.78)	9.74 (0.72)	9.91 (0.62)	9.78 (0.96)	9.54 (0.64)
	Latency (minutes)^i^	19.92 (16.55)	20.98 (14.41)	19.97 (18.05)	16.44 (11.91)	22.19 (11.85)	25.00 (18.03)
**Screen time**		**n=44**	**n=41**	**n=40**	**n=38**	**n=40**	**n=38**
	Week day (hours)^j^	2.52 (2.55)	2.82 (3.87)	1.37 (1.06)	1.73 (2.47)	2.20 (2.91)	1.26 (0.99)
	Weekend day (hours)^j^	2.94 (1.98)	3.15 (2.95)	2.31 (1.56)	1.84 (1.43)	2.68 (2.33)	2.04 (1.39)
**Percentage activity**		**n=34**	**n=35**	**n=27**	**n=31**	**n=26**	**n=27**
	Sedentary time^i^	46.28 (7.98)	47.44 (11.09)	48.28 (7.87)	49.17 (4.03)	46.45 (6.21)	49.47 (5.56)
	Light, moderate, and vigorous physical activity^i^	27.74 (7.40)	25.82 (6.24)	26.18 (6.16)	25.61 (4.38)	27.73 (5.42)	25.44 (4.93)
	Moderate-to-vigorous physical activity^i^	13.88 (5.04)	12.02 (3.60)	13.56 (4.43)	12.91 (3.70)	14.38 (4.11)	13.01 (3.77)

^a^Calculated from 24-hour diet recall using Easy Diet Diary/Foodworks.

^b^n=37.

^c^From food questionnaire.

^d^Scored from food questionnaire questions on frequency of intake of takeaway or fast food; sugary cereals; potato chips or other salty foods; sweets; and cakes, doughnuts, sweet cookies, or muffins. Responses of never or rarely, 1 to 3 times per month, 1 to 2 times per week, 3 to 4 times per week, 5 to 6 times per week, once per day, and 2 or more times per day were coded as 1 to 6, respectively, and summed to obtain a discretionary food score.

^e^Self-efficacy questionnaire.

^f^Child-feeding questionnaire.

^g^Parent modeling questionnaire.

^h^From sleep questionnaire.

^i^Accelerometer measures.

^j^From screen time questionnaire.

**Table 3:** Results of intention-to-treat analyses for primary and secondary outcomes; linear mixed model group × time interaction (random intercept and compound symmetry covariance structure). Age, cohort, and baseline values included as covariates in the model (n=86).

Variable	Estimate	95% CI	*P* value^a^
Body mass index	−0.11	−0.34 to 0.12	.35
kJ/kg of body weight^b^	10.89	−29.94 to 51.73	.60
Percentage of kJ from sugar^b^	−0.09	−2.44 to 2.25	.94
Percentage of kJ from saturated fat^b^	−0.61	−3.09 to 1.87	.63
Servings of fruit^c^	−0.24	−0.58 to 0.10	.17
Servings of vegetables^c^	0.17	−0.15 to 0.49	.24
Discretionary food frequency score^d^	−1.36	−2.27 to −0.45	<.01
Nutrition self-efficacy^e^	0.43	0.10 to 0.76	*.01^f^*
Child feeding–restriction^g^	0.04	−0.21 to 0.29	.76
Child feeding–pressure^g^	−0.30	−0.61 to −0.00	*.048*
Parent modeling^h^	0.21	−0.02 to 0.44	.08
Sleep duration (hours)^i^	−0.22	−0.57 to 0.13	.21
Sleep latency (minutes)^i^	−0.25	−0.79 to 0.74	.95
Sleep reluctance^j^	−0.36	−0.77 to 0.06	.09
Screen time–week day (hours)^k^	−0.20	−0.87 to 0.47	.56
Screen time–weekend day (hours)^k^	−0.40	−0.90 to 0.10	.11
Percentage sedentary time^i^	0.84	−1.60 to −3.27	.49
Percentage light, moderate, and vigorous intensity physical activity^i,k^	−0.99	−2.20 to 2.01	.93
Percentage moderate-to-vigorous intensity physical activity^i^	0.54	−0.94 to 2.01	.47

^a^Significant at *P*<.05.

^b^Calculated from 24-hour diet recall using Easy Diet Diary/Foodworks.

^c^From Food Questionnaire.

^d^Scored from food questionnaire questions on the frequency of intake of takeaway or fast food; sugary cereals; potato chips or other salty foods; sweets; and cakes, doughnuts, sweet cookies, or muffins. Responses of never or rarely, 1 to 3 times per month, 1 to 2 times per week, 3 to 4 times per week, 5 to 6 times per week, once per day, and 2 or more times per day were coded as 1 to 6, respectively, and summed to obtain a discretionary food score.

^e^Self-efficacy questionnaire.

^f^Italicized text: statistically significant result.

^g^Child feeding questionnaire.

^h^Parent modeling questionnaire.

^i^Accelerometer measures.

^j^From sleep questionnaire.

^k^From screen time questionnaire.

**Table 4:** Adjusted mean differences (and 95% CI) for primary and secondary outcomes at 3 months (complete case analyses). Analysis of covariance (ANCOVA) analyses, with baseline value, age, and cohort as covariates (n=78).

Variable	Intervention comparison, adjusted mean difference (95% CI)	*P* value^a^
Body mass index	−0.23 (−0.50 to 0.04)	.09
kJ/kg of body weight^b^	−0.57 (−57.71 to 46.26)	.83
Percentage of kJ from sugar^b^	−0.23 (−3.29 to 2.83)	.88
Percentage of kJ from saturated fat^b^	−0.15 (−1.94 to 1.63)	.87
Servings of fruit^c^	−0.31 (−0.69 to 0.07)	.11
Servings of vegetables^c^	0.19 (−0.23 to 0.60)	.37
Frequency discretionary foods^d^	−1.45 (−2.47 to −0.43)	.01
Nutrition self-efficacy^e^	0.33 (−0.03 to 0.69)	.07
Child feeding–restriction^f^	0.01 (−0.28 to 0.29)	.96
Child feeding–pressure^f^	−0.27 (−0.61 to 0.07)	.12
Parent modeling^g^	0.24 (0.06 to 0.53)	.12
Sleep duration^h^	0.04 (−0.35 to 0.43)	.84
Sleep latency^h^	−4.46 (−13.91 to 4.98)	.35
Sleep reluctance^i^	−0.36 (−0.82 to 0.09)	.11
Screen time–weekday^j^	0.45 (−0.36 to 1.27)	.27
Screen time–weekend^j^	−0.30 (−0.86 to 0.26)	.29
Percentage sedentary time^h^	0.14 (−2.76 to 3.04)	.92
Percentage light, moderate, and vigorous intensity physical activity^h^	0.92 (−1.60 to 3.44)	.47
Percentage moderate-to-vigorous intensity physical activity^h^	1.10 (−0.65 to 2.84)	.21

^a^Significant at *P*<.05.

^b^Calculated from 24-hour diet recall using Easy Diet Diary/Foodworks.

^c^From food questionnaire.

^d^Scored from food questionnaire questions on frequency of intake of takeaway or fast food; sugary cereals; potato chips or other salty foods; sweets; and cakes, doughnuts, sweet cookies, or muffins. Responses of never or rarely, 1 to 3 times per month, 1 to 2 times per week, 3 to 4 times per week, 5 to 6 times per week, once per day, and 2 or more times per day were coded as 1-6, respectively, and summed to obtain a discretionary food score.

^e^Self-efficacy questionnaire.

^f^Child feeding questionnaire.

^g^Parent modeling questionnaire.

^h^Accelerometer measures.

^i^From sleep questionnaire.

^j^From screen time questionnaire.

**Table 5:** Adjusted mean differences (and 95% CI) for primary and secondary outcomes at 6 months (complete case analyses). Analysis of covariance (ANCOVA) analyses, with baseline value, age, and cohort as covariates (n=78).

Variable	Intervention comparison, adjusted mean difference (95% CI)	*P* value^a^
Body mass index	0.01 (−0.27 to −0.29)	.95
kJ/kg of body weight^b^	24.80 (−17.75 to 67.35)	.25
Percentage of kJ from sugar^b^	0.05 (−3.18 to 3.29)	.97
Percentage of kJ from saturated fat^b^	−1.41 (−3.19 to 0.37)	.12
Servings of fruit^c^	−0.17 (−0.57 to 0.23)	.39
Servings of vegetables^c^	0.16 (−0.24 to 0.56)	.44
Frequency discretionary foods^d^	−1.30 (−2.34 to −0.26)	.02
Nutrition self-efficacy^e^	0.53 (0.13 to 0.93)	.01
Child feeding–restriction^f^	0.10 (−0.18 to 0.37)	.48
Child feeding–pressure^f^	−0.35 (−0.68 to −0.02)	.04
Parent modeling^g^	0.18 (−0.05 to 0.41)	.12
Sleep duration^h^	−0.55 (−1.01 to −0.03)	.04
Sleep latency^h^	6.00 (−4.09 to 16.09)	.24
Sleep reluctance^i^	−0.33 (−0.82 to 0.15)	.18
Screen time–weekday^j^	−0.84 (−1.76 to 0.07)	.07
Screen time–weekend^j^	−0.49 (−1.14 to 0.15)	.13
Percentage sedentary time^k^	1.590 (−1.415 to 4.60)	.29
Percentage light, moderate, and vigorous intensity physical activity^h^	−1.106 (−3.601 to 1.40)	.38
Percentage moderate-to-vigorous intensity physical activity^h^	−1.110 (−1.912 to 1.69)	.90

^a^Significant at *P*<.05.

^b^Calculated from 24-hour diet recall using Easy Diet Diary/Foodworks.

^c^From food questionnaire.

^d^Scored from food questionnaire questions on frequency of intake of takeaway or fast food; sugary cereals; potato chips or other salty foods; sweets; and cakes, doughnuts, sweet cookies, or muffins. Responses of never or rarely, 1 to 3 times per month, 1 to 2 times per week, 3 to 4 times per week, 5 to 6 times per week, once per day, and 2 or more times per day were coded as 1-6, respectively, and summed to obtain a discretionary food score.

^e^Self-efficacy questionnaire.

^f^Child feeding questionnaire.

^g^Parent modeling questionnaire.

^h^Accelerometer measures.

^i^From sleep questionnaire.

^j^From screen time questionnaire.

**Table 6:** *Time2bHealthy* intervention process evaluation (n=38).

Question	Strongly agree, n (%)	Agree, n (%)	Neutral, n (%)	Disagree, n (%)	Strongly disagree, n (%)	Not applicable, n (%)
The program content was interesting	21 (55)	15 (39)	2 (5)	0 (0)	0 (0)	0 (0)
The program content was easy to understand	28 (74)	10 (26)	0 (0)	0 (0)	0 (0)	0 (0)
The program content was relevant	22 (58)	15 (39)	1 (3)	0 (0)	0 (0)	0 (0)
The length of the program was appropriate	15 (39)	18 (47)	2 (5)	3 (8)	0 (0)	0 (0)
One module every 2 weeks was appropriate	11 (29)	23 (61)	4 (11)	0 (0)	0 (0)	0 (0)
The tips and tricks for parents was helpful	20 (53)	17 (45)	1 (3)	0 (0)	0 (0)	0 (0)
The information about meals was helpful	22 (58)	13 (34)	2 (5)	1 (3)	0 (0)	0 (0)
There was enough information in the module about meals	14 (37)	20 (53)	0 (0)	4 (11)	0 (0)	0 (0)
The information on snacks and drinks was helpful	21 (55)	15 (39)	1 (3)	1 (3)	0 (0)	0 (0)
There was enough information in the module about snacks and drinks	15 (39)	19 (50)	2 (5)	2 (5)	0 (0)	0 (0)
The information about physical activity was helpful	20 (53)	16 (42)	2 (5)	0 (0)	0 (0)	0 (0)
There was enough information in the module about physical activity	19 (50)	17 (45)	2 (5)	0 (0)	0 (0)	0 (0)
The information on screen time was helpful	20 (53)	13 (34)	4 (11)	1 (3)	0 (0)	0 (0)
There was enough information in the module about screen time	14 (37)	20 (53)	4 (11)	0 (0)	0 (0)	0 (0)
The information about sleep was helpful	11 (29)	19 (50)	6 (16)	0 (0)	1 (3)	1 (3)
There was enough information about sleep	8 (21)	24 (63)	4 (11)	0 (0)	1 (3)	1 (3)
The goal setting was helpful	12 (32)	18 (47)	7 (18)	1 (3)	0 (0)	0 (0)
The number of goals set was appropriate	12 (32)	18 (47)	7 (18)	0 (0)	1 (3)	0 (0)
The health consultants were helpful and knowledgeable	20 (53)	15 (39)	1 (3)	1 (3)	0 (0)	1 (3)
The time the health consultants responded in was appropriate	21 (55)	16 (42)	0 (0)	0 (0)	0 (0)	1 (3)
The online delivery mode was suitable	19 (50)	18 (47)	1 (3)	0 (0)	0 (0)	0 (0)
The Facebook group component was useful	3 (8)	12 (32)	18 (47)	4 (11)	1 (3)	0 (0)

### Process Evaluation

Overall, 38 participants from the intervention group (38/42, 90%) completed the process evaluation questionnaire. The results are displayed in Table 6. Most participants agreed or strongly agreed that the program content was interesting (36/38, 95%), easy to understand (38/38, 100%), and relevant (37/38, 97%). Most also agreed or strongly agreed that the length of the program was appropriate (33/38, 87%), the goal-setting component was helpful (30/38, 79%), and that the dietitian was helpful and knowledgeable (35/38, 92%). Most participants discussed the program with extended family members (28/38, 74%). The internet-based delivery mode of the program was suitable for the majority of participants (37/38, 97%); however, 6 participants stated that they would have preferred a different mode of delivery such as a mobile-optimized website (2) mobile phone app (2), face-to-face (2), or hard copy (2). Only 15 participants (15/38, 39%) agreed or strongly agreed that the Facebook component was useful.

## Discussion

### Principal Findings

In this RCT, we found no significant difference in the BMI change between the 2 groups at 6 months post baseline. There were no significant differences in physical activity, screen time, or sleep outcomes between groups. The intervention did, however, demonstrate some positive group-by-time outcomes in relation to dietary intake, child feeding, and nutrition parent self-efficacy. To the best of our knowledge, *Time2bHealthy* is the first RCT to assess the efficacy of a parent-focused healthy lifestyle intervention on BMI in preschool-aged children, which is delivered entirely Web-based.

Our null finding regarding BMI change at 6 months aligns with similar eHealth obesity prevention studies conducted in young [45] and older children [17,21,46] and a recent mobile health study in preschool-aged children that measured fat mass index [47]. Due to a lack of eHealth studies in this age group, we have also compared our findings with studies delivered by more traditional methods. Mixed results have been reported from traditionally delivered parent-focused obesity prevention studies in young children, with a recent meta-analysis finding a short-term, but not a long-term, effect [48]. This meta-analysis also found that interventions targeting only overweight and obese children were more effective than those that included children in the healthy weight range [48]. Given that more than 90% (78/86) of children recruited in our study were in the healthy weight range, significant changes may have been unrealistic. Superior outcomes may have been achieved had our study included only overweight and obese children. Healthy weight children were included in this study as prevention is key to impacting childhood obesity rates, and it is critical to design interventions that facilitate establishment of healthy behaviors and maintenance of healthy weight in all children at an early age [49]. There was a significant within-group difference in BMI in the intervention group. Had the target sample size been achieved, it is possible that a difference between groups would have been found.

Other eHealth parent-focused studies have demonstrated similar improvements in dietary outcomes, such as energy dense food consumption [50,51]. The discretionary food group-by-time outcomes in this study most closely align with Williamson et al’s [52] internet-based study targeting adolescent overweight girls, which demonstrated a reduction in “eating fattening foods.” Contrary to this study, previous eHealth studies have also shown improvements in fruit and vegetable intake, including Chen et al’s internet-based study on adolescents [21] and Knowlden and Conrad’s internet-based study for mothers of 4- to 6-year-old children [53]. Reduction in sugar-sweetened beverage intake was also reported in an internet-based parent-focused study for children aged 18 to 24 months [54]. Some traditionally delivered parent-focused interventions in preschool-aged children have also demonstrated improvements in fruit and vegetable consumption [55] and reductions in mean energy intake [56].

Our null findings in regard to kJ/kg body weight and kJ from sugar and saturated fat were perhaps due to the fact that (due to resource constraints) the 24-hour recall was administered on 1 single weekday at each time point and was not sufficient to capture regular and weekend consumption patterns. It is also possible that the intervention effects on each of the obesity-related behaviors could have been diluted due to the multi-behavior focus and breadth of the content covered compared with previous studies that have focused on fewer behaviors.

Similar eHealth parent-focused studies in a range of age groups have shown mixed physical activity outcomes [21,46,47,52,57,58]. One successful internet-based study of adolescents used pedometers to self-monitor activity [21], which may have enhanced motivation. Few traditionally delivered parent-focused studies have demonstrated an improvement in physical activity [59]. Accelerometry compliance was not optimal in our study (n=53 to 68), and therefore, the results may not be indicative of the whole sample. Night-time accelerometry compliance was even lower (n=41 to 68). To the best of our knowledge, no similar eHealth studies have assessed sleep outcomes; however, a traditionally delivered program found a significant increase in parent-reported sleep duration [60]. Further studies are needed, which objectively measure sleep duration and explore strategies to improve night-time accelerometry compliance, such as the use of wrist-worn monitors [61], incentives, or phone calls/email reminders [62]. Screen time behavior has also not been a focus of many parent-focused childhood obesity studies. One eHealth study in young children [45] and 2 in older children found null screen time outcomes [20,46], which align with our findings. Similar to our study, Knowlden et al [53] found improvements in both groups and an improvement in screen time parent self-efficacy in the intervention group; perhaps, a minimal intervention can effect change in this area.

There was a significant group-by-time interaction for nutrition parent self-efficacy but no significant differences between the intervention and comparison groups for parent self-efficacy in relation to physical activity, screen time, or sleep. The reason why positive outcomes were achieved for nutrition parent self-efficacy and not for the other behaviors is unclear, but it may be due to the higher proportion of program time dedicated to healthy eating and nutrition (2 modules compared with only 1 module for the other behaviors) and the larger number of videos, providing a greater opportunity for vicarious learning.

It is established that parent self-efficacy is crucial for implementing obesity-related behavior change in children [44]. Positive relationships have been reported between high parental (or maternal) self-efficacy and fruit and vegetable intake [63-66] and moderate-to-vigorous intensity physical activity [65], and an inverse relationship has been reported with consumption of unhealthy food [63,65]. Although social cognitive theory has been used as a basis for other similar studies [17,20], parent self-efficacy has rarely been assessed, despite this being key in parent-focused interventions.

There was a significant group-by-time interaction for “pressure-to-eat” child feeding practices, but there was no significant difference between groups for “restriction” of child-feeding practices. Despite the body of evidence regarding child feeding practices and risk of overweight and obesity, there are limited studies that have used child feeding as an outcome measure. No other eHealth study to the best of our knowledge has assessed child feeding practices; hence, the outcomes of this study will be compared with traditionally delivered programs in preschool-aged children. Similar to this study, a significant improvement in “pressure-to-eat” child feeding practices was reported in a group that received a regular newsletter (compared with a group that received a single booklet), but no significant changes in other child-feeding practices were reported in a study of mothers of African American preschool-aged children [67]. Conversely, Harvey-Berino et al [50] found a significant reduction in “restriction” child-feeding practices, but not for other child-feeding practices in their childhood obesity prevention study in Native American preschool children, which was delivered in the home [50]. As most studies have reported a significant change in only 1 child-feeding practice, it is possible that in this study as well as others, parents may find it difficult to focus on changing more than 1 of the practices simultaneously.

Research clearly demonstrates the need to intervene early to establish healthy behaviors [68], and the role of parents at this stage is instrumental in achieving change [4,7,49,69]. The results of this RCT suggest that an internet-based program can be effective in facilitating change, particularly for dietary-related behaviors, and weight status range of children in this sample demonstrates that the intervention can be applied to both healthy weight and overweight/obese children. The positive dietary-related outcomes may be a reflection of a higher proportion of the program being focused on healthy eating and the activities in these modules being more intensive and involving more practical application. The dietary-related modules were also completed first, with 32 (76.19%) participants completing these 2 modules. Participation (and perhaps motivation) dropped off as participants worked through the modules, with 26 participants (61.90%) completing all the 6 modules.

A cost-effectiveness analysis was not within the scope of this study. Although it is generally perceived that eHealth interventions are more cost-effective than traditionally delivered programs, more research is needed [70].

Recruitment for this study was challenging, despite the expansion of the recruitment area and extension of the recruitment period, and we are not able to determine with certainty the factors involved in the lower than anticipated sample size without further investigation. Further work is required to explore optimal avenues to access at-risk and hard-to-reach populations. The program was marketed as a “healthy lifestyle program” and appeared to be more successful in recruiting parents of children in the healthy weight range than overweight or obese ranges. Parental awareness of their child’s weight status may have been a factor in the low enrollment rates in the overweight and obese ranges. Previous research has found that the majority of parents do not recognize that their child is overweight [71], and therefore, parents may not have recognized the need for the program. Education and monitoring initiatives may, therefore, be useful to enhance parent awareness. Feedback from participants who initially enquired about the study indicated that the need to attend face-to-face appointments for data collection was a deterrent. As the intervention is solely internet-based, it could be easily translated to a real-world setting, given that most developed countries [72-74] have a high proportion of internet users. In a real-world setting, data collection could be Web-based, which could improve participant recruitment and retention, but lack of objectively measured data may create bias issues. The requirement for participants to have a Facebook account may also have been a factor if potential participants did not have an interest in engaging with social media or felt uncomfortable sharing information online with people they did not know. It is recommended that further studies with a longer follow-up period and those that translate programs into primary health care be conducted to demonstrate long-term effectiveness.

### Strengths and Limitations

This study used a randomized controlled design, applying a backwards intervention mapping exercise to align the intervention with social cognitive theory [29,30]. Multiple health behaviors were targeted, and outcome measures were based on objective and valid methods where possible. There was a low attrition rate, and the mode of delivery, content, and format of the program demonstrated a high rate of user acceptability.

There are several limitations of this study. Although it was intentional to include healthy weight children in this study, there were a higher than anticipated proportion of children (over 90%, 78/86) in the healthy weight range. Therefore, the effect on BMI may have been diluted. Due to the small number of children in the overweight and obese ranges, it was not possible to conduct a subanalysis of these participants. Statistical power would have been affected by the fact that the target sample size was not achieved despite measures to enhance participant recruitment, including expanding the recruitment area and extending the recruitment period. It is also possible that a longer follow-up period may have been required to demonstrate differences in BMI change between groups. As there were multiple outcomes assessed, there is a risk that there may have been a type 1 error. Questionnaire-based measures and the 24-hour recall used for secondary outcomes, involving self-reporting of data, were used, and therefore, it may have been possible that parents misreported this information (either intentionally or unintentionally) and such misreporting would probably have occurred in both groups. This is a familiar challenge to researchers assessing behavioral outcomes [75,76]. A height measure could not be obtained at the data collection appointment for 2 participants. Parent- provided measures were used in these instances.

In conclusion, *Time2bHealthy* led to a significant improvement in the frequency of discretionary food intake, nutrition parent self-efficacy, and pressure-to-eat child-feeding practices, but no improvement in BMI. The program has the potential for scalability and wide reach. Future studies with a larger sample size and longer follow-up period and those that translate effective eHealth childhood obesity prevention programs into primary health care are needed.

## Appendix

Multimedia Appendix 1 CONSORT‐EHEALTH checklist (V 1.6.1).

## Abbreviations

ANCOVA: analysis of covariance
ANOVA: analysis of variance
BMI: body mass index
eHealth: electronic health
ITT: intention-to-treat
RCT: randomized controlled trial
WHO: World Health Organization
