# Supplementary material for: An Internet-Based Childhood Obesity Prevention Program (Time2bHealthy) for Parents of Preschool-Aged Children: Randomized Controlled Trial
Source: J Med Internet Res. 2019 Feb 8;21(2):e11964. doi: 10.2196/11964 (PMC6384541; doi:10.2196/11964)
Supplement: Multimedia Appendix 1 [file jmir_v21i2e11964_app1.pdf]

CONSORT-EHEALTH (V 1.6.1) - Submission/Publication Form

The CONSORT-EHEALTH checklist is intended for authors of randomized trials evaluating web-based and Internet-based applications/interventions, including mobile interventions, electronic games (incl multiplayer games), social media, certain telehealth applications, and other interactive and/or networked electronic applications. Some of the items (e.g. all subitems under item 5 - description of the intervention) may also be applicable for other study designs.

The goal of the CONSORT EHEALTH checklist and guideline is to be
a) a guide for reporting for authors of RCTs,
b) to form a basis for appraisal of an ehealth trial (in terms of validity)

CONSORT-EHEALTH items/subitems are MANDATORY reporting items for studies published in the Journal of Medical Internet Research and other journals / scientific societies endorsing the checklist.

Items numbered 1., 2., 3., 4a., 4b etc are original CONSORT or CONSORT-NPT (non-pharmacologic treatment) items.
Items with Roman numerals (i., ii, iii, iv etc.) are CONSORT-EHEALTH extensions/clarifications.

As the CONSORT-EHEALTH checklist is still considered in a formative stage, we would ask that you also RATE ON A SCALE OF 1-5 how important/useful you feel each item is FOR THE PURPOSE OF THE CHECKLIST and reporting guideline (optional).

Mandatory reporting items are marked with a red *.
In the textboxes, either copy & paste the relevant sections from your manuscript into this form - please include any quotes from your manuscript in QUOTATION MARKS,
or answer directly by providing additional information not in the manuscript, or elaborating on why the item was not relevant for this study.

YOUR ANSWERS WILL BE PUBLISHED AS A SUPPLEMENTARY FILE TO YOUR PUBLICATION IN JMIR AND ARE CONSIDERED PART OF YOUR PUBLICATION (IF ACCEPTED).
Please fill in these questions diligently. Information will not be copyedited, so please use proper spelling and grammar, use correct capitalization, and avoid abbreviations.

DO NOT FORGET TO SAVE AS PDF _AND_ CLICK THE SUBMIT BUTTON SO YOUR ANSWERS ARE IN OUR DATABASE !!!

Citation Suggestion (if you append the pdf as Appendix we suggest to cite this paper in the caption):
Eysenbach G, CONSORT-EHEALTH Group
CONSORT-EHEALTH: Improving and Standardizing Evaluation Reports of Web-based and Mobile Health Interventions
J Med Internet Res 2011;13(4):e126
URL: http://www.jmir.org/2011/4/e126/
doi: 10.2196/jmir.1923
PMID: 22209829

*Required

Your name *

First Last

Megan Hammersley

Primary Affiliation (short), City, Country *

University of Toronto, Toronto, Canada

University of Wollongong, Wollongong, A

Your e-mail address *

abc@gmail.com

mlh965@uowmail.edu.au

Title of your manuscript *

Provide the (draft) title of your manuscript.

Time2bHealthy – an internet-based childhood obesity prevention program for parents of preschool-aged children: outcomes of a randomized controlled trial

Name of your App/Software/Intervention *

If there is a short and a long/alternate name, write the short name first and add the long name in brackets.

Time2bHealthy

Evaluated Version (if any)

e.g. "V1", "Release 2017-03-01", "Version 2.0.27913"

Your answer

Language(s) *

What language is the **intervention**/app in? If multiple languages are available, separate by comma (e.g. "English, French")

English

URL of your Intervention Website or App

e.g. a direct link to the mobile app on app in appstore (itunes, Google Play), or URL of the website. If the **intervention** is a DVD or hardware, you can also link to an Amazon page.

https://time2bhealthy.uow.edu.au/

URL of an image/screenshot (optional)

Your answer

Accessibility *

Can an enduser access the **intervention** presently?

- ( ) access is free and open
- ( ) access only for special usergroups, not open
- ( ) access is open to everyone, but requires payment/subscription/in-app purchases
- (x) app/**intervention** no longer accessible
- ( ) Other:

Primary Medical Indication/Disease/Condition *

e.g. "Stress", "Diabetes", or define the target group in brackets after the condition, e.g. "Autism (Parents of children with)", "Alzheimers (Informal Caregivers of)"

Childhood Obesity

Primary Outcomes measured in trial *

comma-separated list of primary outcomes reported in the trial

Body Mass Index

Secondary/other outcomes

Are there any other outcomes the **intervention** is expected to affect?

Dietary intake, physical activity, screen-time, sleep, child feeding, parent self-efficacy, parental modelling

Recommended "Dose" *

What do the instructions for users say on how often the app should be used?

- ○ Approximately Daily
- ◉ Approximately Weekly
- ○ Approximately Monthly
- ○ Approximately Yearly
- ○ "as needed"
- ○ Other:

Approx. Percentage of Users (starters) still using the app as recommended after 3 months *

- ○ unknown / not evaluated
- ○ 0-10%
- ○ 11-20%
- ○ 21-30%
- ○ 31-40%
- ○ 41-50%
- ○ 51-60%
- ◉ 61-70%
- ○ 71%-80%
- ○ 81-90%
- ○ 91-100%
- ○ Other:

Overall, was the app/intervention effective? *

- ( ) yes: all primary outcomes were significantly better in **intervention** group vs control
- ( ) partly: SOME primary outcomes were significantly better in **intervention** group vs control
- ( ) no statistically significant difference between control and **intervention**
- ( ) potentially harmful: control was significantly better than **intervention** in one or more outcomes
- ( ) inconclusive: more research is needed
- (•) Other: No statistically significant difference between control and interventic

Article Preparation Status/Stage *

At which stage in your article preparation are you currently (at the time you fill in this form)

- ( ) not submitted yet - in early draft status
- (•) not submitted yet - in late draft status, just before submission
- ( ) submitted to a journal but not reviewed yet
- ( ) submitted to a journal and after receiving initial reviewer comments
- ( ) submitted to a journal and accepted, but not published yet
- ( ) published
- ( ) Other:

Journal *

If you already know where you will submit this paper (or if it is already submitted), please provide the journal name (if it is not JMIR, provide the journal name under "other")

- ○ not submitted yet / unclear where I will submit this
- ◉ Journal of Medical Internet Research (JMIR)
- ○ JMIR mHealth and UHealth
- ○ JMIR Serious Games
- ○ JMIR Mental Health
- ○ JMIR Public Health
- ○ JMIR Formative Research
- ○ Other JMIR sister journal
- ○ Other:

Is this a full powered effectiveness trial or a pilot/feasibility trial? *

- ○ Pilot/feasibility
- ◉ Fully powered

Manuscript tracking number *

If this is a JMIR submission, please provide the manuscript tracking number under "other" (The ms tracking number can be found in the submission acknowledgement email, or when you login as author in JMIR. If the paper is already published in JMIR, then the ms tracking number is the four-digit number at the end of the DOI, to be found at the bottom of each published article in JMIR)

- ◉ no ms number (yet) / not (yet) submitted to / published in JMIR
- ○ Other:

TITLE AND ABSTRACT

1a) TITLE: Identification as a randomized trial in the title

1a) Does your paper address CONSORT item 1a? *

I.e does the title contain the phrase "Randomized Controlled Trial"? (if not, explain the reason under "other")

- (selected) yes
- Other:

1a-i) Identify the mode of delivery in the title

Identify the mode of delivery. Preferably use "web-based" and/or "mobile" and/or "electronic game" in the title. Avoid ambiguous terms like "online", "virtual", "interactive". Use "Internet-based" only if Intervention includes non-web-based Internet components (e.g. email), use "computer-based" or "electronic" only if offline products are used. Use "virtual" only in the context of "virtual reality" (3-D worlds). Use "online" only in the context of "online support groups". Complement or substitute product names with broader terms for the class of products (such as "mobile" or "smart phone" instead of "iphone"), especially if the application runs on different platforms.

	1	2	3	4	5	
subitem not at all important	○	○	○	●	○	essential

Does your paper address subitem 1a-i? *

Copy and paste relevant sections from manuscript title (include quotes in quotation marks "like this" to indicate direct quotes from your manuscript), or elaborate on this item by providing additional information not in the ms, or briefly explain why the item is not applicable/relevant for your study

"

1a-ii) Non-web-based components or important co-interventions in title

Mention non-web-based components or important co-interventions in title, if any (e.g., "with telephone support").

	1	2	3	4	5	
subitem not at all important	○	○	○	●	○	essential

Does your paper address subitem 1a-ii?

Copy and paste relevant sections from manuscript title (include quotes in quotation marks "like this" to indicate direct quotes from your manuscript), or elaborate on this item by providing additional information not in the ms, or briefly explain why the item is not applicable/relevant for your study

N/A

1a-iii) Primary condition or target group in the title

Mention primary condition or target group in the title, if any (e.g., “for children with Type I Diabetes”) Example: A Web-based and Mobile Intervention with Telephone Support for Children with Type I Diabetes: Randomized Controlled Trial

	1	2	3	4	5	
subitem not at all important	○	○	○	○	●	essential

Does your paper address subitem 1a-iii? *

Copy and paste relevant sections from manuscript title (include quotes in quotation marks "like this" to indicate direct quotes from your manuscript), or elaborate on this item by providing additional information not in the ms, or briefly explain why the item is not applicable/relevant for your study

"Time2bHealthy – an internet-based childhood obesity prevention program for parents of preschool-aged children: outcomes of a randomized controlled trial"

1b) ABSTRACT: Structured summary of trial design, methods, results, and conclusions

NPT extension: Description of experimental treatment, comparator, care providers, centers, and blinding status.

1b-i) Key features/functionalities/components of the intervention and comparator in the METHODS section of the ABSTRACT

Mention key features/functionalities/components of the intervention and comparator in the abstract. If possible, also mention theories and principles used for designing the site. Keep in mind the needs of systematic reviewers and indexers by including important synonyms. (Note: Only report in the abstract what the main paper is reporting. If this information is missing from the main body of text, consider adding it)

	1	2	3	4	5	
subitem not at all important	○	○	○	●	○	essential

Does your paper address subitem 1b-i? *

Copy and paste relevant sections from the manuscript abstract (include quotes in quotation marks "like this" to indicate direct quotes from your manuscript), or elaborate on this item by providing additional information not in the ms, or briefly explain why the item is not applicable/relevant for your study

"Intervention participants received an 11-week internet-based healthy lifestyle program, which was underpinned by Social Cognitive Theory, followed by fortnightly emails for 3-months thereafter. Comparison participants received email communication only"

1b-ii) Level of human involvement in the METHODS section of the ABSTRACT

Clarify the level of human involvement in the abstract, e.g., use phrases like “fully automated” vs. “therapist/nurse/care provider/physician-assisted” (mention number and expertise of providers involved, if any). (Note: Only report in the abstract what the main paper is reporting. If this information is missing from the main body of text, consider adding it)

	1	2	3	4	5	
subitem not at all important	○	○	●	○	○	essential

Does your paper address subitem 1b-ii?

Copy and paste relevant sections from the manuscript abstract (include quotes in quotation marks "like this" to indicate direct quotes from your manuscript), or elaborate on this item by providing additional information not in the ms, or briefly explain why the item is not applicable/relevant for your study

"Intervention participants were encouraged to access and contribute to a closed (secret) Facebook group to communicate with other participants and a dietitian".

1b-iii) Open vs. closed, web-based (self-assessment) vs. face-to-face assessments in the METHODS section of the ABSTRACT

Mention how participants were recruited (online vs. offline), e.g., from an open access website or from a clinic or a closed online user group (closed usergroup trial), and clarify if this was a purely web-based trial, or there were face-to-face components (as part of the intervention or for assessment). Clearly say if outcomes were self-assessed through questionnaires (as common in web-based trials). Note: In traditional offline trials, an open trial (open-label trial) is a type of clinical trial in which both the researchers and participants know which treatment is being administered. To avoid confusion, use “blinded” or “unblinded” to indicated the level of blinding instead of “open”, as “open” in web-based trials usually refers to “open access” (i.e. participants can self-enrol). (Note: Only report in the abstract what the main paper is reporting. If this information is missing from the main body of text, consider adding it)

	1	2	3	4	5	
subitem not at all important	○	○	○	◉	○	essential

Does your paper address subitem 1b-iii?

Copy and paste relevant sections from the manuscript abstract (include quotes in quotation marks "like this" to indicate direct quotes from your manuscript), or elaborate on this item by providing additional information not in the ms, or briefly explain why the item is not applicable/relevant for your study

"Participants were recruited both online and through more traditional means". "All data were collected at face-to-face appointments at baseline, 3- and 6-months by blinded data collectors".

1b-iv) RESULTS section in abstract must contain use data

Report number of participants enrolled/assessed in each group, the use/uptake of the intervention (e.g., attrition/adherence metrics, use over time, number of logins etc.), in addition to primary/secondary outcomes. (Note: Only report in the abstract what the main paper is reporting. If this information is missing from the main body of text, consider adding it)

	1	2	3	4	5	
subitem not at all important	○	○	○	○	◉	essential

Does your paper address subitem 1b-iv?

Copy and paste relevant sections from the manuscript abstract (include quotes in quotation marks "like this" to indicate direct quotes from your manuscript), or elaborate on this item by providing additional information not in the ms, or briefly explain why the item is not applicable/relevant for your study

"Eighty-six dyads were recruited, with 42 randomized to the intervention group and 44 to the comparison group. 78 dyads attended the 3- and 6-month follow-ups, with 7 lost to follow-up and one withdrawing". "Sixty-nine percent of participants completed at least 5 of the 6 modules".

1b-v) CONCLUSIONS/DISCUSSION in abstract for negative trials

Conclusions/Discussions in abstract for negative trials: Discuss the primary outcome - if the trial is negative (primary outcome not changed), and the **intervention** was not used, discuss whether negative results are attributable to lack of uptake and discuss reasons. (Note: Only report in the abstract what the main paper is reporting. If this information is missing from the main body of text, consider adding it)

	1	2	3	4	5	
subitem not at all important	○	○	○	○	●	essential

Does your paper address subitem 1b-v?

Copy and paste relevant sections from the manuscript abstract (include quotes in quotation marks "like this" to indicate direct quotes from your manuscript), or elaborate on this item by providing additional information not in the ms, or briefly explain why the item is not applicable/relevant for your study

"The target sample size was not achieved, which would have affected statistical power".

INTRODUCTION

2a) In INTRODUCTION: Scientific background and explanation of rationale

2a-i) Problem and the type of system/solution

Describe the problem and the type of system/solution that is object of the study: intended as stand-alone **intervention** vs. incorporated in broader health care program? Intended for a particular patient population? Goals of the **intervention**, e.g., being more cost-effective to other **interventions**, replace or complement other solutions? (Note: Details about the **intervention** are provided in "Methods" under 5)

	1	2	3	4	5	
subitem not at all important	○	○	○	○	●	essential

Does your paper address subitem 2a-i? *

Copy and paste relevant sections from the manuscript (include quotes in quotation marks "like this" to indicate direct quotes from your manuscript), or elaborate on this item by providing additional information not in the ms, or briefly explain why the item is not applicable/relevant for your study

"The World Health Organization (WHO) has described childhood obesity as one of the most significant public health issues. Around 23% of children and adolescents in developed countries and 13% in developing countries are overweight or obese. One of the main influences on the development of childhood obesity is parental guidance and role-modelling around obesity-related behaviors, particularly in the early years of life up to 5 years of age. Health behaviors become more difficult to change with age and tend to track into adulthood, but are quite malleable in the early years. Therefore, early childhood is an opportune time to intervene and involving parents in interventions appears to be crucial".

"Overweight and obesity interventions which use an eHealth delivery method offer many advantages compared to traditional delivery methods, particularly around convenience and accessibility".

"In this review, no studies targeting children under the age of five years were included and it was recommended that larger, higher quality parent-focussed eHealth studies be conducted, with a particular focus on younger age groups".

"This paper reports the outcomes of a randomized controlled trial (RCT) evaluating the efficacy of a parent-focussed internet-based program in facilitating behavior change in preschool-aged children who are overweight or at-risk of becoming overweight".

2a-ii) Scientific background, rationale: What is known about the (type of) system

Scientific background, rationale: What is known about the (type of) system that is the object of the study (be sure to discuss the use of similar systems for other conditions/diagnoses, if appropiate), motivation for the study, i.e. what are the reasons for and what is the context for this specific study, from which stakeholder viewpoint is the study performed, potential impact of findings [2]. Briefly justify the choice of the comparator.

	1	2	3	4	5	
subitem not at all important	○	○	○	◉	○	essential

Does your paper address subitem 2a-ii? *

Copy and paste relevant sections from the manuscript (include quotes in quotation marks "like this" to indicate direct quotes from your manuscript), or elaborate on this item by providing additional information not in the ms, or briefly explain why the item is not applicable/relevant for your study

"Overweight and obesity interventions which use an eHealth delivery method offer many advantages compared to traditional delivery methods, particularly around convenience and accessibility".
"In this review, no studies targeting children under the age of five years were included and it was recommended that larger, higher quality parent-focussed eHealth studies be conducted, with a particular focus on younger age groups".
"This paper reports the outcomes of a randomized controlled trial (RCT) evaluating the efficacy of a parent-focussed internet-based program in facilitating behavior change in preschool-aged children who are overweight or at-risk of becoming overweight".
"We hypothesized that children in the intervention group would achieve significantly greater reductions in BMI compared to the comparison group at 6-month follow-up. It was also hypothesized that the intervention group would achieve significantly greater improvements in child dietary intake, physical activity, screen-time, sleep, child feeding and parent self-efficacy and role modelling".

2b) In INTRODUCTION: Specific objectives or hypotheses

Does your paper address CONSORT subitem 2b? *

Copy and paste relevant sections from the manuscript (include quotes in quotation marks "like this" to indicate direct quotes from your manuscript), or elaborate on this item by providing additional information not in the ms, or briefly explain why the item is not applicable/relevant for your study

"We hypothesized that children in the intervention group would achieve significantly greater reductions in BMI compared to the comparison group at 6-month follow-up. It was also hypothesized that the intervention group would achieve significantly greater improvements in child dietary intake, physical activity, screen-time, sleep, child feeding and parent self-efficacy and role modelling".

METHODS

3a) Description of trial design (such as parallel, factorial) including allocation ratio

Does your paper address CONSORT subitem 3a? *

Copy and paste relevant sections from the manuscript (include quotes in quotation marks "like this" to indicate direct quotes from your manuscript), or elaborate on this item by providing additional information not in the ms, or briefly explain why the item is not applicable/relevant for your study

"The current study was a two-arm parallel RCT involving parent-child dyads, recruited into six cohorts".
"Randomization was performed in a 1:1 ratio using a concealed computerized random number generator".

3b) Important changes to methods after trial commencement (such as eligibility criteria), with reasons

Does your paper address CONSORT subitem 3b? *

Copy and paste relevant sections from the manuscript (include quotes in quotation marks "like this" to indicate direct quotes from your manuscript), or elaborate on this item by providing additional information not in the ms, or briefly explain why the item is not applicable/relevant for your study

Not applicable. There were no changes to the methods after trial commencement.

3b-i) Bug fixes, Downtimes, Content Changes

Bug fixes, Downtimes, Content Changes: ehealth systems are often dynamic systems. A description of changes to methods therefore also includes important changes made on the **intervention** or comparator during the trial (e.g., major bug fixes or changes in the functionality or content) (5-iii) and other “unexpected events” that may have influenced study design such as staff changes, system failures/downtimes, etc. [2].

	1	2	3	4	5	
subitem not at all important	○	○	○	○	◉	essential

Does your paper address subitem 3b-i?

Copy and paste relevant sections from the manuscript (include quotes in quotation marks "like this" to indicate direct quotes from your manuscript), or elaborate on this item by providing additional information not in the ms, or briefly explain why the item is not applicable/relevant for your study

Not applicable. There were no bug fixes, down times or content changes. There was a previous feasibility trial which ensured that there were no issues such as these in this RCT.

4a) Eligibility criteria for participants

Does your paper address CONSORT subitem 4a? *

Copy and paste relevant sections from the manuscript (include quotes in quotation marks "like this" to indicate direct quotes from your manuscript), or elaborate on this item by providing additional information not in the ms, or briefly explain why the item is not applicable/relevant for your study

"Participants were eligible if their child was 2-5 years of age (and not yet attending school) and was at or above the WHO 50th percentile for body mass index (BMI) for their age and sex [24, 25]. Parents also needed to have a Facebook account or agreed to create one.
Child participants were excluded if they were taking medications or had a medical condition with the potential to affect weight or restrict age-appropriate play. Children with conditions which required the restriction of certain foods (e.g. Coeliac Disease or food allergies) were deemed eligible to participate, but parents were informed that parts of the program would not be completely appropriate and that they would need to make some adaptations to the material provided in order to match their child’s individual dietary/health needs".

4a-i) Computer / Internet literacy

Computer / Internet literacy is often an implicit “de facto” eligibility criterion - this should be explicitly clarified.

	1	2	3	4	5	
subitem not at all important	○	○	○	◉	○	essential

Does your paper address subitem 4a-i?

Copy and paste relevant sections from the manuscript (include quotes in quotation marks "like this" to indicate direct quotes from your manuscript), or elaborate on this item by providing additional information not in the ms, or briefly explain why the item is not applicable/relevant for your study

"Participants were eligible if they had access to the internet.." Access to the internet was determined, rather than computer/internet literacy.

4a-ii) Open vs. closed, web-based vs. face-to-face assessments:

Open vs. closed, web-based vs. face-to-face assessments: Mention how participants were recruited (online vs. offline), e.g., from an open access website or from a clinic, and clarify if this was a purely web-based trial, or there were face-to-face components (as part of the intervention or for assessment), i.e., to what degree got the study team to know the participant. In online-only trials, clarify if participants were quasi-anonymous and whether having multiple identities was possible or whether technical or logistical measures (e.g., cookies, email confirmation, phone calls) were used to detect/prevent these.

	1	2	3	4	5	
subitem not at all important	○	○	○	○	◉	essential

Does your paper address subitem 4a-ii? *

Copy and paste relevant sections from the manuscript (include quotes in quotation marks "like this" to indicate direct quotes from your manuscript), or elaborate on this item by providing additional information not in the ms, or briefly explain why the item is not applicable/relevant for your study

"Provisional eligibility was determined through contact with participants via phone or email and was confirmed at the face-to-face baseline data collection visit when the child's height and weight were measured to confirm if the child's BMI was at or above the WHO 50th percentile for age and sex".
"Participant measures were collected at the University of Wollongong, in the participant's home or in a community setting. Questionnaires were completed by the parents on an iPad during these sessions, which took approximately 30-45 minutes. Demographic information was also collected from parents at the baseline data collection point. Participants in the intervention group were asked to complete an evaluation questionnaire at the end of the online program, which assessed user acceptability of the program content, length, goal-setting, Facebook discussion group and the modality used".

4a-iii) Information giving during recruitment

Information given during recruitment. Specify how participants were briefed for recruitment and in the informed consent procedures (e.g., publish the informed consent documentation as appendix, see also item X26), as this information may have an effect on user self-selection, user expectation and may also bias results.

	1	2	3	4	5	
subitem not at all important	○	○	○	○	◉	essential

Does your paper address subitem 4a-iii?

Copy and paste relevant sections from the manuscript (include quotes in quotation marks "like this" to indicate direct quotes from your manuscript), or elaborate on this item by providing additional information not in the ms, or briefly explain why the item is not applicable/relevant for your study

"Informed written consent was provided by the parents/guardians after reading a participant information sheet".

4b) Settings and locations where the data were collected

Does your paper address CONSORT subitem 4b? *

Copy and paste relevant sections from the manuscript (include quotes in quotation marks "like this" to indicate direct quotes from your manuscript), or elaborate on this item by providing additional information not in the ms, or briefly explain why the item is not applicable/relevant for your study

"Measurements were taken at baseline, 3- and 6-months post-baseline. Participant measures were collected at the University of Wollongong, in the participant's home or in a community setting. Questionnaires were completed by the parents on an iPad during these sessions, which took approximately 30-45 minutes. Demographic information was also collected from parents at the baseline data collection point. Participants in the intervention group were asked to complete an evaluation questionnaire at the end of the online program, which assessed user acceptability of the program content, length, goal-setting, Facebook discussion group and the modality used".

4b-i) Report if outcomes were (self-)assessed through online questionnaires

Clearly report if outcomes were (self-)assessed through online questionnaires (as common in web-based trials) or otherwise.

	1	2	3	4	5	
subitem not at all important	○	○	○	○	◉	essential

Does your paper address subitem 4b-i? *

Copy and paste relevant sections from the manuscript (include quotes in quotation marks "like this" to indicate direct quotes from your manuscript), or elaborate on this item by providing additional information not in the ms, or briefly explain why the item is not applicable/relevant for your study

"Participants in the intervention group were asked to complete an evaluation questionnaire at the end of the online program, which assessed user acceptability of the program content, length, goal-setting, Facebook discussion group and the modality used".

4b-ii) Report how institutional affiliations are displayed

Report how institutional affiliations are displayed to potential participants [on ehealth media], as affiliations with prestigious hospitals or universities may affect volunteer rates, use, and reactions with regards to an intervention.(Not a required item – describe only if this may bias results)

	1	2	3	4	5	
subitem not at all important	○	○	○	◉	○	essential

Does your paper address subitem 4b-ii?

Copy and paste relevant sections from the manuscript (include quotes in quotation marks "like this" to indicate direct quotes from your manuscript), or elaborate on this item by providing additional information not in the ms, or briefly explain why the item is not applicable/relevant for your study

Not reported in manuscript. Details of all investigators and affiliations were provided on the participant information sheet.

5) The **intervention**s for each group with sufficient details to allow replication, including how and when they were actually administered

5-i) Mention names, credential, affiliations of the developers, sponsors, and owners

Mention names, credential, affiliations of the developers, sponsors, and owners [6] (if authors/evaluators are owners or developer of the software, this needs to be declared in a "Conflict of interest" section or mentioned elsewhere in the manuscript).

	1	2	3	4	5	
subitem not at all important	○	○	○	○	◉	essential

Does your paper address subitem 5-i?

Copy and paste relevant sections from the manuscript (include quotes in quotation marks "like this" to indicate direct quotes from your manuscript), or elaborate on this item by providing additional information not in the ms, or briefly explain why the item is not applicable/relevant for your study

Conflict of Interest: "The University of Wollongong developed the Time2bHealthy Program".

5-ii) Describe the history/development process

Describe the history/development process of the application and previous formative evaluations (e.g., focus groups, usability testing), as these will have an impact on adoption/use rates and help with interpreting results.

	1	2	3	4	5	
subitem not at all important	○	○	○	○	◉	essential

Does your paper address subitem 5-ii?

Copy and paste relevant sections from the manuscript (include quotes in quotation marks "like this" to indicate direct quotes from your manuscript), or elaborate on this item by providing additional information not in the ms, or briefly explain why the item is not applicable/relevant for your study

"The protocol for this study has been published. Briefly, the Time2bHealthy study was based on formative research with parents of preschool-aged children and was piloted prior to this trial".

5-iii) Revisions and updating

Revisions and updating. Clearly mention the date and/or version number of the application/intervention (and comparator, if applicable) evaluated, or describe whether the intervention underwent major changes during the evaluation process, or whether the development and/or content was "frozen" during the trial. Describe dynamic components such as news feeds or changing content which may have an impact on the replicability of the intervention (for unexpected events see item 3b).

	1	2	3	4	5	
subitem not at all important	○	○	○	◉	○	essential

Does your paper address subitem 5-iii?

Copy and paste relevant sections from the manuscript (include quotes in quotation marks "like this" to indicate direct quotes from your manuscript), or elaborate on this item by providing additional information not in the ms, or briefly explain why the item is not applicable/relevant for your study

Details regarding changes made to the intervention are provided in the previously published protocol paper. Hammersley ML, Jones RA, and Okely AD, Time2bHealthy - An online childhood obesity prevention program for preschool-aged children: A randomised controlled trial protocol. Contemp Clin Trials 2017; 61:73-80.
One module was added to the program after the feasibility trial. Amendments were made to ensure consistency with latest guidelines and enhancements made such as addition of videos.

5-iv) Quality assurance methods

Provide information on quality assurance methods to ensure accuracy and quality of information provided [1], if applicable.

	1	2	3	4	5	
subitem not at all important	○	○	◉	○	○	essential

Does your paper address subitem 5-iv?

Copy and paste relevant sections from the manuscript (include quotes in quotation marks "like this" to indicate direct quotes from your manuscript), or elaborate on this item by providing additional information not in the ms, or briefly explain why the item is not applicable/relevant for your study

Content was consistent with evidence-based guidelines

5-v) Ensure replicability by publishing the source code, and/or providing screenshots/screen-capture video, and/or providing flowcharts of the algorithms used

Ensure replicability by publishing the source code, and/or providing screenshots/screen-capture video, and/or providing flowcharts of the algorithms used. Replicability (i.e., other researchers should in principle be able to replicate the study) is a hallmark of scientific reporting.

	1	2	3	4	5	
subitem not at all important	○	○	◉	○	○	essential

Does your paper address subitem 5-v?

Copy and paste relevant sections from the manuscript (include quotes in quotation marks "like this" to indicate direct quotes from your manuscript), or elaborate on this item by providing additional information not in the ms, or briefly explain why the item is not applicable/relevant for your study

Should the manuscript be accepted for publication, we will discuss provision of screen-shots of the program. We are currently planning to implement a translational research trial involving a few external stakeholders, so we will need to consider the level of information that can be published.

5-vi) Digital preservation

Digital preservation: Provide the URL of the application, but as the **intervention** is likely to change or disappear over the course of the years; also make sure the **intervention** is archived (Internet Archive, webcitation.org, and/or publishing the source code or screenshots/videos alongside the article). As pages behind login screens cannot be archived, consider creating demo pages which are accessible without login.

	1	2	3	4	5	
subitem not at all important	○	○	○	○	○	essential

Does your paper address subitem 5-vi?

Copy and paste relevant sections from the manuscript (include quotes in quotation marks "like this" to indicate direct quotes from your manuscript), or elaborate on this item by providing additional information not in the ms, or briefly explain why the item is not applicable/relevant for your study

The current website for the program is https://time2bhealthy.uow.edu.au/ however the content does sit behind a login. As discussed in 5-v, should the manuscript be accepted for publication, we will discuss the provision of screen-shots.

5-vii) Access

Access: Describe how participants accessed the application, in what setting/context, if they had to pay (or were paid) or not, whether they had to be a member of specific group. If known, describe how participants obtained “access to the platform and Internet” [1]. To ensure access for editors/reviewers/readers, consider to provide a “backdoor” login account or demo mode for reviewers/readers to explore the application (also important for archiving purposes, see vi).

	1	2	3	4	5	
subitem not at all important	○	○	○	◉	○	essential

Does your paper address subitem 5-vii? *

Copy and paste relevant sections from the manuscript (include quotes in quotation marks "like this" to indicate direct quotes from your manuscript), or elaborate on this item by providing additional information not in the ms, or briefly explain why the item is not applicable/relevant for your study

"Participants randomized to the intervention group were provided with an individual login to access the Time2bHealthy program"
"The intervention targeted multiple behaviors and consisted of six modules including an introduction, nutrition (n=2), physical activity, screen-time and sleep which were completed by participants over an 11-week period. Each module comprised of reading material, videos, activities, quizzes and a goal-setting component. Participants received feedback on their goals at the end of each module by a dietitian and were provided with advice to improve their goals using the SMART goal framework. Participants also received weekly emails reminding them to log on to the website and participate in the activities. Participants in each of the cohorts were encouraged to access and contribute to a closed (secret) Facebook group to communicate with other members of the cohort and the dietitian. There was a separate group for each cohort and they were regularly monitored and moderated by the dietitian. Participants were asked to post photos, recipes and personal experiences and ideas that they had found helpful for behavior change which were relevant to each module".
"Participants continued to receive emails fortnightly at the end of the program until the 6-month follow-up. Infographics summarising the key points from each of the modules were provided in these emails and participants were also encouraged to log back into the website to revise the material and review their progress with their goals".

5-viii) Mode of delivery, features/functionalities/components of the intervention and comparator, and the theoretical framework

Describe mode of delivery, features/functionalities/components of the intervention and comparator, and the theoretical framework [6] used to design them (instructional strategy [1], behaviour change techniques, persuasive features, etc., see e.g., [7, 8] for terminology). This includes an in-depth description of the content (including where it is coming from and who developed it) [1],” whether [and how] it is tailored to individual circumstances and allows users to track their progress and receive feedback” [6]. This also includes a description of communication delivery channels and – if computer-mediated communication is a component – whether communication was synchronous or asynchronous [6]. It also includes information on presentation strategies [1], including page design principles, average amount of text on pages, presence of hyperlinks to other resources, etc. [1].

	1	2	3	4	5	
subitem not at all important	○	○	○	◉	○	essential

Does your paper address subitem 5-viii? *

Copy and paste relevant sections from the manuscript (include quotes in quotation marks "like this" to indicate direct quotes from your manuscript), or elaborate on this item by providing additional information not in the ms, or briefly explain why the item is not applicable/relevant for your study

"The development, content and theoretical framework for this intervention has been previously published. Briefly, the intervention was guided by Bandura's Social Cognitive Theory and was designed using a backwards intervention mapping process. The intervention targeted multiple behaviors and consisted of six modules including an introduction, nutrition (n=2), physical activity, screen-time and sleep which were completed by participants over an 11-week period. Each module comprised of reading material, videos, activities, quizzes and a goal-setting component. Participants received feedback on their goals at the end of each module by a dietitian and were provided with advice to improve their goals using the SMART goal framework. Participants also received weekly emails reminding them to log on to the website and participate in the activities. Participants in each of the cohorts were encouraged to access and contribute to a closed (secret) Facebook group to communicate with other members of the cohort and the dietitian"
"Participants continued to receive emails fortnightly at the end of the program until the 6-month follow-up. Infographics summarising the key points from each of the modules were provided in these emails and participants were also encouraged to log back into the website to revise the material and review their progress with their goals".
Further details are also provided in the protocol paper: Hammersley ML, Jones RA, and Okely AD, Time2bHealthy - An online childhood obesity prevention program for preschool-aged children: A randomised controlled trial protocol. Contemp Clin Trials 2017; 61:73-80.

5-ix) Describe use parameters

Describe use parameters (e.g., intended "doses" and optimal timing for use). Clarify what instructions or recommendations were given to the user, e.g., regarding timing, frequency, heaviness of use, if any, or was the **intervention** used ad libitum.

	1	2	3	4	5	
subitem not at all important	○	○	○	●	○	essential

Does your paper address subitem 5-ix?

Copy and paste relevant sections from the manuscript (include quotes in quotation marks "like this" to indicate direct quotes from your manuscript), or elaborate on this item by providing additional information not in the ms, or briefly explain why the item is not applicable/relevant for your study

"The intervention targeted multiple behaviors and consisted of six modules including an introduction, nutrition (n=2), physical activity, screen-time and sleep which were completed by participants over an 11-week period". Participants received weekly emails to remind them to log on and they were guided to complete one module per fortnight.
"Participants continued to receive emails fortnightly at the end of the program until the 6-month follow-up".

5-x) Clarify the level of human involvement

Clarify the level of human involvement (care providers or health professionals, also technical assistance) in the e-intervention or as co-intervention (detail number and expertise of professionals involved, if any, as well as “type of assistance offered, the timing and frequency of the support, how it is initiated, and the medium by which the assistance is delivered”. It may be necessary to distinguish between the level of human involvement required for the trial, and the level of human involvement required for a routine application outside of a RCT setting (discuss under item 21 – generalizability).

	1	2	3	4	5	
subitem not at all important	○	○	○	●	○	essential

Does your paper address subitem 5-x?

Copy and paste relevant sections from the manuscript (include quotes in quotation marks "like this" to indicate direct quotes from your manuscript), or elaborate on this item by providing additional information not in the ms, or briefly explain why the item is not applicable/relevant for your study

"Participants received feedback on their goals at the end of each module by a dietitian and were provided with advice to improve their goals using the SMART goal framework. Participants also received weekly emails reminding them to log on to the website and participate in the activities. Participants in each of the cohorts were encouraged to access and contribute to a closed (secret) Facebook group to communicate with other members of the cohort and the dietitian".

5-xi) Report any prompts/reminders used

Report any prompts/reminders used: Clarify if there were prompts (letters, emails, phone calls, SMS) to use the application, what triggered them, frequency etc. It may be necessary to distinguish between the level of prompts/reminders required for the trial, and the level of prompts/reminders for a routine application outside of a RCT setting (discuss under item 21 – generalizability).

	1	2	3	4	5	
subitem not at all important	○	○	○	○	◉	essential

Does your paper address subitem 5-xi? *

Copy and paste relevant sections from the manuscript (include quotes in quotation marks "like this" to indicate direct quotes from your manuscript), or elaborate on this item by providing additional information not in the ms, or briefly explain why the item is not applicable/relevant for your study

"Participants also received weekly emails reminding them to log on to the website and participate in the activities".

5-xii) Describe any co-interventions (incl. training/support)

Describe any co-interventions (incl. training/support): Clearly state any interventions that are provided in addition to the targeted eHealth intervention, as ehealth intervention may not be designed as stand-alone intervention. This includes training sessions and support [1]. It may be necessary to distinguish between the level of training required for the trial, and the level of training for a routine application outside of a RCT setting (discuss under item 21 – generalizability.

	1	2	3	4	5	
subitem not at all important	○	○	○	◉	○	essential

Does your paper address subitem 5-xii? *

Copy and paste relevant sections from the manuscript (include quotes in quotation marks "like this" to indicate direct quotes from your manuscript), or elaborate on this item by providing additional information not in the ms, or briefly explain why the item is not applicable/relevant for your study

Not applicable. There were no co-interventions.

6a) Completely defined pre-specified primary and secondary outcome measures, including how and when they were assessed

Does your paper address CONSORT subitem 6a? *

Copy and paste relevant sections from the manuscript (include quotes in quotation marks "like this" to indicate direct quotes from your manuscript), or elaborate on this item by providing additional information not in the ms, or briefly explain why the item is not applicable/relevant for your study

"Child height and weight were measured using a standardized method [30] to calculate BMI. A stadiometer was used to measure height to the nearest 0.1mm. Weight was measured (with no shoes and minimal clothing) to the nearest 0.1kg using a SECA scale. Both height and weight were measured twice. The mean of these two measurements was used to calculate BMI. A third measurement was taken when height measurements differed by more than 0.5cm and weight measurements differed by more than 0.5kg."
"Dietary intake was assessed using both a parent-reported food questionnaire (modified from the Eating and Physical Activity Questionnaire (EPAQ) and a parent-reported 24-h recall of child dietary intake (using the 'Easy Diet Diary' app (Xyris Software (Australia) Pty Ltd)). The section of the food questionnaire which asked about frequency of intake of discretionary foods was expanded to include additional discretionary food categories, which used the same scale as the existing question. Cronbach's alpha=0.68 for these discretionary food questions. Data from the 24-h recall was used to calculate kJ per kg of body weight, percentage of kJ from sugar and percentage of kJ from saturated fat. Data from the food questionnaire were used to assess daily fruit intake, daily vegetable intake and frequency of fruit juice and sugary drinks intake. A discretionary food score was calculated based on responses to questions on frequency of intake of takeaway or fast food; sugary cereals; potato chips or other salty snacks; sweets; cakes, doughnuts, and sweet cookies or muffins".
"Physical activity intensity and duration was measured using an ActiGraph GT3X+ accelerometer (ActiGraph Corporation, Pensacola, FL) which was worn on an elasticized belt around the child's waist for seven days. Accelerometer data were analysed in ActiLife version 6 (ActiGraph Corporation, Pensacola, FL). A sampling frequency of 30 Hz was used, with the files then reintegrated into 15 s epochs. Non-wear time was defined as 20 minutes or more of 0 counts. Accelerometer data used for the physical activity analysis were considered valid based on wear time of at least 6 hours per day on 3 days, which has been found to be reliable in previous research. The following cut-points appropriate for preschool-aged children were used to categorize physical activity intensity; sedentary <100 counts/min, low light-intensity physical activity 101-800 counts/min, high light-intensity physical activity 801-1679 counts/min, moderate-intensity physical activity 1680-3367 count/min and vigorous-intensity physical activity ≥1680 count/min".
"Sleep habits were assessed using four questions assessing sleep latency, sleep reluctance, difficulty sleeping and falling to sleep in own bed based on questions from the Children's Sleep Habits Questionnaire (Sneddon et al 2013) (Cronbach's alpha=0.63 for the three scaled questions relating to sleep reluctance, difficulty

falling asleep and falling to sleep in own bed), questions about the child's usual sleep and wake times and an Actigraph GT3X+ accelerometer. Sleep accelerometer data were analysed in ActiLife using the Sadeh algorithm, which is appropriate for use in children. Sleep accelerometer data were considered valid based on wear time of at least 3 nights".
"Parent-reported questionnaires were used to assess child feeding (from the Child Feeding Questionnaire pre-defined subscales of 'restriction' and 'pressure to eat'), screen-time (based on [37, 38] and additional questions relating to screen entertainment rules, presence of a TV in the child's bedroom and frequency of watching TV while eating a meal), parent modelling (developed after reviewing, Cronbach's alpha=0.63), and parent self-efficacy in nutrition, physical activity, screen-time and sleep (modified from Bohman et al, 2013 by adding six additional questions and making small changes to some existing questions to align the questionnaire to the program content. Cronbach's alpha=0.89)".

6a-i) Online questionnaires: describe if they were validated for online use and apply CHERRIES items to describe how the questionnaires were designed/deployed

If outcomes were obtained through online questionnaires, describe if they were validated for online use and apply CHERRIES items to describe how the questionnaires were designed/deployed [9].

	1	2	3	4	5	
subitem not at all important	○	○	○	◉	○	essential

Does your paper address subitem 6a-i?

Copy and paste relevant sections from manuscript text

Questionnaires used for outcome measures were not used online, they were completed by participants on an iPad in a face-to-face setting.

6a-ii) Describe whether and how "use" (including intensity of use/dosage) was defined/measured/monitored

Describe whether and how "use" (including intensity of use/dosage) was defined/measured/monitored (logins, logfile analysis, etc.). Use/adoption metrics are important process outcomes that should be reported in any ehealth trial.

	1	2	3	4	5	
subitem not at all important	○	○	○	◉	○	essential

Does your paper address subitem 6a-ii?

Copy and paste relevant sections from manuscript text

Use was measured by the number of modules completed. "Within the intervention group, 29 participants (69%) completed at least 5 of the 6 online modules".

6a-iii) Describe whether, how, and when qualitative feedback from participants was obtained

Describe whether, how, and when qualitative feedback from participants was obtained (e.g., through emails, feedback forms, interviews, focus groups).

	1	2	3	4	5	
subitem not at all important	○	○	○	○	◉	essential

Does your paper address subitem 6a-iii?

Copy and paste relevant sections from manuscript text

"Participants in the intervention group were asked to complete a process evaluation questionnaire at the end of the online program, which assessed user acceptability of the program content, length, goal-setting, Facebook discussion group and the modality used".

6b) Any changes to trial outcomes after the trial commenced, with reasons

Does your paper address CONSORT subitem 6b? *

Copy and paste relevant sections from the manuscript (include quotes in quotation marks "like this" to indicate direct quotes from your manuscript), or elaborate on this item by providing additional information not in the ms, or briefly explain why the item is not applicable/relevant for your study

Not applicable. There were no changed to trial outcomes after the trial commenced.

7a) How sample size was determined

NPT: When applicable, details of whether and how the clustering by care provides or centers was addressed

7a-i) Describe whether and how expected attrition was taken into account when calculating the sample size

Describe whether and how expected attrition was taken into account when calculating the sample size.

	1	2	3	4	5	
subitem not at all important	○	○	○	○	◉	essential

Does your paper address subitem 7a-i?

Copy and paste relevant sections from manuscript title (include quotes in quotation marks "like this" to indicate direct quotes from your manuscript), or elaborate on this item by providing additional information not in the ms, or briefly explain why the item is not applicable/relevant for your study

"Based on the results of the pilot study, we expected a BMI effect size of approximately 0.4 for this trial. To detect a statistically significant difference between groups (α=0.05 and power=0.8), 136 participants were required (68 per group) and based on an estimated attrition rate of 15%, we aimed to recruit 160 participants (80 per group)".

7b) When applicable, explanation of any interim analyses and stopping guidelines

Does your paper address CONSORT subitem 7b? *

Copy and paste relevant sections from the manuscript (include quotes in quotation marks "like this" to indicate direct quotes from your manuscript), or elaborate on this item by providing additional information not in the ms, or briefly explain why the item is not applicable/relevant for your study

Not applicable

8a) Method used to generate the random allocation sequence

NPT: When applicable, how care providers were allocated to each trial group

Does your paper address CONSORT subitem 8a? *

Copy and paste relevant sections from the manuscript (include quotes in quotation marks "like this" to indicate direct quotes from your manuscript), or elaborate on this item by providing additional information not in the ms, or briefly explain why the item is not applicable/relevant for your study

"Participants were randomized into the intervention or comparison group following the collection of baseline measures. Randomization was performed in a 1:1 ratio using a concealed computerized random number generator. A data manager with no other involvement in the study conducted the randomization. The researcher responsible for implementing the intervention was the only person who was informed about group allocation. At the follow-up data collection time-points, height and weight measurements were taken by trained data collectors blinded to group allocation".

8b) Type of randomisation; details of any restriction (such as blocking and block size)

Does your paper address CONSORT subitem 8b? *

Copy and paste relevant sections from the manuscript (include quotes in quotation marks "like this" to indicate direct quotes from your manuscript), or elaborate on this item by providing additional information not in the ms, or briefly explain why the item is not applicable/relevant for your study

"Randomization was performed in a 1:1 ratio using a concealed computerized random number generator".

9) Mechanism used to implement the random allocation sequence (such as sequentially numbered containers), describing any steps taken to conceal the sequence until interventions were assigned

Does your paper address CONSORT subitem 9? *

Copy and paste relevant sections from the manuscript (include quotes in quotation marks "like this" to indicate direct quotes from your manuscript), or elaborate on this item by providing additional information not in the ms, or briefly explain why the item is not applicable/relevant for your study

"Randomization was performed in a 1:1 ratio using a concealed computerized random number generator".
"A data manager with no other involvement in the study conducted the randomization. The researcher responsible for implementing the intervention was the only person who was informed about group allocation. At the follow-up data collection time-points, height and weight measurements were taken by trained data collectors blinded to group allocation".

10) Who generated the random allocation sequence, who enrolled participants, and who assigned participants to interventions

Does your paper address CONSORT subitem 10? *

Copy and paste relevant sections from the manuscript (include quotes in quotation marks "like this" to indicate direct quotes from your manuscript), or elaborate on this item by providing additional information not in the ms, or briefly explain why the item is not applicable/relevant for your study

"A data manager with no other involvement in the study conducted the randomization. The researcher responsible for implementing the intervention was the only person who was informed about group allocation". The PhD researcher (dietitian) fielded enquiries, assessed eligibility and enrolled participants into the study.

11a) If done, who was blinded after assignment to interventions (for example, participants, care providers, those assessing outcomes) and how

NPT: Whether or not administering co-**interventions** were blinded to group assignment

11a-i) Specify who was blinded, and who wasn’t

Specify who was blinded, and who wasn’t. Usually, in web-based trials it is not possible to blind the participants [1, 3] (this should be clearly acknowledged), but it may be possible to blind outcome assessors, those doing data analysis or those administering co-**interventions** (if any).

	1	2	3	4	5	
subitem not at all important	○	○	○	○	◉	essential

Does your paper address subitem 11a-i? *

Copy and paste relevant sections from the manuscript (include quotes in quotation marks "like this" to indicate direct quotes from your manuscript), or elaborate on this item by providing additional information not in the ms, or briefly explain why the item is not applicable/relevant for your study

Participants were not blinded and the PhD student researcher (dietitian) was not blinded. Baseline measured were collected by the PhD researcher prior to randomization and follow-up data collection was performed by trained, blinded data collectors.
"Participants were randomized into the intervention or comparison group following the collection of baseline measures. Randomization was performed in a 1:1 ratio using a concealed computerized random number generator. A data manager with no other involvement in the study conducted the randomization. The researcher responsible for implementing the intervention was the only person who was informed about group allocation. At the follow-up data collection time-points, height and weight measurements were taken by trained data collectors blinded to group allocation".

11a-ii) Discuss e.g., whether participants knew which intervention was the “intervention of interest” and which one was the “comparator”

Informed consent procedures (4a-ii) can create biases and certain expectations - discuss e.g., whether participants knew which **intervention** was the “**intervention** of interest” and which one was the “comparator”.

	1	2	3	4	5	
subitem not at all important	○	○	○	◉	○	essential

Does your paper address subitem 11a-ii?

Copy and paste relevant sections from the manuscript (include quotes in quotation marks "like this" to indicate direct quotes from your manuscript), or elaborate on this item by providing additional information not in the ms, or briefly explain why the item is not applicable/relevant for your study

In order to provide informed consent, the information sheet specified the names of the intervention (Time2bHealthy) and comparison (Raising Children) groups. However, at the time of group allocation, participants were informed of the groups they were allocated to, but the names of the groups were referred to (rather than 'intervention' and 'control').

11b) If relevant, description of the similarity of interventions

(this item is usually not relevant for ehealth trials as it refers to similarity of a placebo or sham intervention to a active medication/intervention)

Does your paper address CONSORT subitem 11b? *

Copy and paste relevant sections from the manuscript (include quotes in quotation marks "like this" to indicate direct quotes from your manuscript), or elaborate on this item by providing additional information not in the ms, or briefly explain why the item is not applicable/relevant for your study

Not applicable

12a) Statistical methods used to compare groups for primary and secondary outcomes

NPT: When applicable, details of whether and how the clustering by care providers or centers was addressed

Does your paper address CONSORT subitem 12a? *

Copy and paste relevant sections from the manuscript (include quotes in quotation marks "like this" to indicate direct quotes from your manuscript), or elaborate on this item by providing additional information not in the ms, or briefly explain why the item is not applicable/relevant for your study

"Differences in changes over time between the intervention and comparison groups were assessed for each outcome. Linear mixed models were used to determine differences between groups over time (baseline, 3-months and 6-months) with adjustment for potential covariates. Intention-to-treat (ITT) principles were used for parametric data, with all participants analysed in the group which they were randomized regardless of whether they attended all data collection time-points or completed the intervention. Covariates included baseline values, age and cohort. In addition to the ITT analysis, a completers analysis was conducted using linear mixed models, which included intervention participants who had completed at least 5 modules (>80% of online content) and comparison participants who attended all data collection time-points. Due to non-parametric distributions for some variables, Freidman's tests and Wilcoxon Signed Rank tests were used followed by Mann Whitney tests to analyse non-parametric data using completed cases. Generalized estimating equations were considered, however the analyses would not converge.
Posthoc ANCOVA analyses were used to detect changes between groups at individual time-points, which included the baseline value, age and cohort as covariates. Within group changes were analysed using repeated measures ANOVA, which included age and cohort as covariates. These were complete case analyses. Analyses were performed using IBM SPSS Statistics for Windows, version 25 (IBM Corp., Armonk, N.Y., USA)".

12a-i) Imputation techniques to deal with attrition / missing values

Imputation techniques to deal with attrition / missing values: Not all participants will use the intervention/comparator as intended and attrition is typically high in ehealth trials. Specify how participants who did not use the application or dropped out from the trial were treated in the statistical analysis (a complete case analysis is strongly discouraged, and simple imputation techniques such as LOCF may also be problematic [4]).

	1	2	3	4	5	
subitem not at all important	○	○	○	○	◉	essential

Does your paper address subitem 12a-i? *

Copy and paste relevant sections from the manuscript (include quotes in quotation marks "like this" to indicate direct quotes from your manuscript), or elaborate on this item by providing additional information not in the ms, or briefly explain why the item is not applicable/relevant for your study

Linear mixed models were used to conduct intention-to-treat analyses.
"Linear mixed models were used to determine differences between groups over time (baseline, 3-months and 6-months) with adjustment for potential covariates. Intention-to-treat (ITT) principles were used for parametric data, with all participants analysed in the group which they were randomized regardless of whether they attended all data collection time-points or completed the intervention".

12b) Methods for additional analyses, such as subgroup analyses and adjusted analyses

Does your paper address CONSORT subitem 12b? *

Copy and paste relevant sections from the manuscript (include quotes in quotation marks "like this" to indicate direct quotes from your manuscript), or elaborate on this item by providing additional information not in the ms, or briefly explain why the item is not applicable/relevant for your study

Analyses were adjusted for covariates. "Covariates included baseline values, age and cohort".

X26) REB/IRB Approval and Ethical Considerations [recommended as subheading under "Methods"] (not a CONSORT item)

X26-i) Comment on ethics committee approval

	1	2	3	4	5	
subitem not at all important	○	○	○	○	◉	essential

Does your paper address subitem X26-i?

Copy and paste relevant sections from the manuscript (include quotes in quotation marks "like this" to indicate direct quotes from your manuscript), or elaborate on this item by providing additional information not in the ms, or briefly explain why the item is not applicable/relevant for your study

"The study was registered with the Australian and New Zealand Clinical Trials Registry (12616000119493) and approved by the University of Wollongong Human Research Ethics Committee (HE15/354)".

x26-ii) Outline informed consent procedures

Outline informed consent procedures e.g., if consent was obtained offline or online (how? Checkbox, etc.?), and what information was provided (see 4a-ii). See [6] for some items to be included in informed consent documents.

	1	2	3	4	5	
subitem not at all important	○	○	○	○	●	essential

Does your paper address subitem X26-ii?

Copy and paste relevant sections from the manuscript (include quotes in quotation marks "like this" to indicate direct quotes from your manuscript), or elaborate on this item by providing additional information not in the ms, or briefly explain why the item is not applicable/relevant for your study

"Informed written consent was provided by the parents/guardians after reading a participant information sheet". Information sheets were emailed or given to participants. Consent was not provided online. Hard copy consent forms were collected at the baseline data collection visit.

X26-iii) Safety and security procedures

Safety and security procedures, incl. privacy considerations, and any steps taken to reduce the likelihood or detection of harm (e.g., education and training, availability of a hotline)

	1	2	3	4	5	
subitem not at all important	○	○	○	○	●	essential

Does your paper address subitem X26-iii?

Copy and paste relevant sections from the manuscript (include quotes in quotation marks "like this" to indicate direct quotes from your manuscript), or elaborate on this item by providing additional information not in the ms, or briefly explain why the item is not applicable/relevant for your study

"Participants were informed that they could make contact via email or phone if they had questions or concerns at any time".

RESULTS

13a) For each group, the numbers of participants who were randomly assigned, received intended treatment, and were analysed for the primary outcome

NPT: The number of care providers or centers performing the intervention in each group and the number of patients treated by each care provider in each center

Does your paper address CONSORT subitem 13a? *

Copy and paste relevant sections from the manuscript (include quotes in quotation marks "like this" to indicate direct quotes from your manuscript), or elaborate on this item by providing additional information not in the ms, or briefly explain why the item is not applicable/relevant for your study

"Of the 93 parent/child dyads who attended the initial visit, 86 were eligible and enrolled in the study. Forty-two participant dyads were randomized to the intervention group and 44 to the comparison group. Seventy-eight participants (91%) attended the 3- and 6-month follow-ups, with 7 (8%) lost to follow-up and one participant (1%) withdrawing from the intervention group due to problems accessing the internet". Also see Figure 1.

13b) For each group, losses and exclusions after randomisation, together with reasons

Does your paper address CONSORT subitem 13b? (NOTE: Preferably, this is shown in a CONSORT flow diagram) *

Copy and paste relevant sections from the manuscript (include quotes in quotation marks "like this" to indicate direct quotes from your manuscript), or elaborate on this item by providing additional information not in the ms, or briefly explain why the item is not applicable/relevant for your study

See Figure 1

13b-i) Attrition diagram

Strongly recommended: An attrition diagram (e.g., proportion of participants still logging in or using the intervention/comparator in each group plotted over time, similar to a survival curve) or other figures or tables demonstrating usage/dose/engagement.

	1	2	3	4	5	
subitem not at all important	○	○	○	○	◉	essential

Does your paper address subitem 13b-i?

Copy and paste relevant sections from the manuscript or cite the figure number if applicable (include quotes in quotation marks "like this" to indicate direct quotes from your manuscript), or elaborate on this item by providing additional information not in the ms, or briefly explain why the item is not applicable/relevant for your study

See Figure 2

14a) Dates defining the periods of recruitment and follow-up

Does your paper address CONSORT subitem 14a? *

Copy and paste relevant sections from the manuscript (include quotes in quotation marks "like this" to indicate direct quotes from your manuscript), or elaborate on this item by providing additional information not in the ms, or briefly explain why the item is not applicable/relevant for your study

"Recruitment was conducted between January 2016 and June 2017". "Follow-up was conducted between July 2016 and December 2017".

14a-i) Indicate if critical "secular events" fell into the study period

Indicate if critical "secular events" fell into the study period, e.g., significant changes in Internet resources available or "changes in computer hardware or Internet delivery resources"

	1	2	3	4	5	
subitem not at all important	○	○	○	◉	○	essential

Does your paper address subitem 14a-i?

Copy and paste relevant sections from the manuscript (include quotes in quotation marks "like this" to indicate direct quotes from your manuscript), or elaborate on this item by providing additional information not in the ms, or briefly explain why the item is not applicable/relevant for your study

Not applicable

14b) Why the trial ended or was stopped (early)

Does your paper address CONSORT subitem 14b? *

Copy and paste relevant sections from the manuscript (include quotes in quotation marks "like this" to indicate direct quotes from your manuscript), or elaborate on this item by providing additional information not in the ms, or briefly explain why the item is not applicable/relevant for your study

Not applicable

15) A table showing baseline demographic and clinical characteristics for each group

NPT: When applicable, a description of care providers (case volume, qualification, expertise, etc.) and centers (volume) in each group

Does your paper address CONSORT subitem 15? *

Copy and paste relevant sections from the manuscript (include quotes in quotation marks "like this" to indicate direct quotes from your manuscript), or elaborate on this item by providing additional information not in the ms, or briefly explain why the item is not applicable/relevant for your study

Please refer to Table 1

15-i) Report demographics associated with digital divide issues

In ehealth trials it is particularly important to report demographics associated with digital divide issues, such as age, education, gender, social-economic status, computer/Internet/ehealth literacy of the participants, if known.

	1	2	3	4	5	
subitem not at all important	○	○	○	◉	○	essential

Does your paper address subitem 15-i? *

Copy and paste relevant sections from the manuscript (include quotes in quotation marks "like this" to indicate direct quotes from your manuscript), or elaborate on this item by providing additional information not in the ms, or briefly explain why the item is not applicable/relevant for your study

Please refer to Table 1

16) For each group, number of participants (denominator) included in each analysis and whether the analysis was by original assigned groups

16-i) Report multiple "denominators" and provide definitions

Report multiple "denominators" and provide definitions: Report N's (and effect sizes) "across a range of study participation [and use] thresholds" [1], e.g., N exposed, N consented, N used more than x times, N used more than y weeks, N participants "used" the intervention/comparator at specific pre-defined time points of interest (in absolute and relative numbers per group). Always clearly define "use" of the intervention.

	1	2	3	4	5	
subitem not at all important	○	○	○	○	◉	essential

Does your paper address subitem 16-i? *

Copy and paste relevant sections from the manuscript (include quotes in quotation marks "like this" to indicate direct quotes from your manuscript), or elaborate on this item by providing additional information not in the ms, or briefly explain why the item is not applicable/relevant for your study

Refer to Figure 1 for number of participants included at each page. Refer to Tables for number of participants included in analyses. Note: main analysis used intention-to-treat principles.

16-ii) Primary analysis should be intent-to-treat

Primary analysis should be intent-to-treat, secondary analyses could include comparing only “users”, with the appropriate caveats that this is no longer a randomized sample (see 18-i).

	1	2	3	4	5	
subitem not at all important	○	○	○	○	◉	essential

Does your paper address subitem 16-ii?

Copy and paste relevant sections from the manuscript (include quotes in quotation marks "like this" to indicate direct quotes from your manuscript), or elaborate on this item by providing additional information not in the ms, or briefly explain why the item is not applicable/relevant for your study

"Intention-to-treat (ITT) principles were used for parametric data, with all participants analysed in the group which they were randomized regardless of whether they attended all data collection time-points or completed the intervention".

17a) For each primary and secondary outcome, results for each group, and the estimated effect size and its precision (such as 95% confidence interval)

Does your paper address CONSORT subitem 17a? *

Copy and paste relevant sections from the manuscript (include quotes in quotation marks "like this" to indicate direct quotes from your manuscript), or elaborate on this item by providing additional information not in the ms, or briefly explain why the item is not applicable/relevant for your study

Main results for each group, precision and significance are provided in table 2.

17a-i) Presentation of process outcomes such as metrics of use and intensity of use

In addition to primary/secondary (clinical) outcomes, the presentation of process outcomes such as metrics of use and intensity of use (dose, exposure) and their operational definitions is critical. This does not only refer to metrics of attrition (13-b) (often a binary variable), but also to more continuous exposure metrics such as “average session length”. These must be accompanied by a technical description how a metric like a “session” is defined (e.g., timeout after idle time) [1] (report under item 6a).

	1	2	3	4	5	
subitem not at all important	○	○	○	◉	○	essential

Does your paper address subitem 17a-i?

Copy and paste relevant sections from the manuscript (include quotes in quotation marks "like this" to indicate direct quotes from your manuscript), or elaborate on this item by providing additional information not in the ms, or briefly explain why the item is not applicable/relevant for your study

"Figure 2 shows the completion of each of the intervention program modules. At least 5 of 6 modules were completed by 29 participants (69%)".

17b) For binary outcomes, presentation of both absolute and relative effect sizes is recommended

Does your paper address CONSORT subitem 17b? *

Copy and paste relevant sections from the manuscript (include quotes in quotation marks "like this" to indicate direct quotes from your manuscript), or elaborate on this item by providing additional information not in the ms, or briefly explain why the item is not applicable/relevant for your study

Not applicable. There were no binary outcomes included in the analyses.

18) Results of any other analyses performed, including subgroup analyses and adjusted analyses, distinguishing pre-specified from exploratory

Does your paper address CONSORT subitem 18? *

Copy and paste relevant sections from the manuscript (include quotes in quotation marks "like this" to indicate direct quotes from your manuscript), or elaborate on this item by providing additional information not in the ms, or briefly explain why the item is not applicable/relevant for your study

There were no sub-group analyses, but ANCOVA analyses were conducted to determine differences between groups at each time-point and repeated measures ANOVA analyses were conducted to determine change within groups. "The ANCOVA analyses (shown in Table 3) also found no significant differences between groups at each time-point. When considering changes within groups, the repeated measures ANOVA found a significant change in BMI within the intervention group at both the 3-month (adjusted mean difference -0.262, 95% CI -0.506 to -0.017, p=0.032) and 6-month time-point (adjusted mean difference -0.216, 95% CI -0.401 to -0.031, p=0.017) and no significant changes within the comparison group (as displayed in Table 4)".

18-i) Subgroup analysis of comparing only users

A subgroup analysis of comparing only users is not uncommon in ehealth trials, but if done, it must be stressed that this is a self-selected sample and no longer an unbiased sample from a randomized trial (see 16-iii).

	1	2	3	4	5	
subitem not at all important	○	○	○	◉	○	essential

Does your paper address subitem 18-i?

Copy and paste relevant sections from the manuscript (include quotes in quotation marks "like this" to indicate direct quotes from your manuscript), or elaborate on this item by providing additional information not in the ms, or briefly explain why the item is not applicable/relevant for your study

"A completers analysis was conducted using linear mixed models, which included intervention participants who had completed at least 5 modules (>80% of online content) and comparison participants who attended all data collection time-points".

19) All important harms or unintended effects in each group

(for specific guidance see CONSORT for harms)

Does your paper address CONSORT subitem 19? *

Copy and paste relevant sections from the manuscript (include quotes in quotation marks "like this" to indicate direct quotes from your manuscript), or elaborate on this item by providing additional information not in the ms, or briefly explain why the item is not applicable/relevant for your study

Not applicable

19-i) Include privacy breaches, technical problems

Include privacy breaches, technical problems. This does not only include physical “harm” to participants, but also incidents such as perceived or real privacy breaches [1], technical problems, and other unexpected/unintended incidents. “Unintended effects” also includes unintended positive effects [2].

	1	2	3	4	5	
subitem not at all important	○	○	○	○	◉	essential

Does your paper address subitem 19-i?

Copy and paste relevant sections from the manuscript (include quotes in quotation marks "like this" to indicate direct quotes from your manuscript), or elaborate on this item by providing additional information not in the ms, or briefly explain why the item is not applicable/relevant for your study

Not applicable

19-ii) Include qualitative feedback from participants or observations from staff/researchers

Include qualitative feedback from participants or observations from staff/researchers, if available, on strengths and shortcomings of the application, especially if they point to unintended/unexpected effects or uses. This includes (if available) reasons for why people did or did not use the application as intended by the developers.

	1	2	3	4	5	
subitem not at all important	○	○	○	○	◉	essential

Does your paper address subitem 19-ii?

Copy and paste relevant sections from the manuscript (include quotes in quotation marks "like this" to indicate direct quotes from your manuscript), or elaborate on this item by providing additional information not in the ms, or briefly explain why the item is not applicable/relevant for your study

A process evaluation was completed, but this did not determine any unexpected or unexpected effects of uses. "Thirty-seven participants from the intervention group (88%) completed the process evaluation questionnaire". "Most participants agreed or strongly agreed that the program content was interesting (95%), easy to understand (100%) and relevant (97%). Most also agreed or strongly agreed that the length of the program was appropriate (87%), the goal-setting component was helpful (79%) and that the dietitian was helpful and knowledgeable (92%). Most participants discussed the program with extended family members (74%). The internet-based delivery mode of the program was suitable for the majority of participants (97%), however six participants stated that they would have preferred a different mode of delivery such as a mobile-optimized website (2), smartphone app (2), face-to-face (2) or hard copy (2). Only 15 participants (41%) agreed or strongly agreed that the Facebook component was useful".

DISCUSSION

22) Interpretation consistent with results, balancing benefits and harms, and considering other relevant evidence

NPT: In addition, take into account the choice of the comparator, lack of or partial blinding, and unequal expertise of care providers or centers in each group

22-i) Restate study questions and summarize the answers suggested by the data, starting with primary outcomes and process outcomes (use)

Restate study questions and summarize the answers suggested by the data, starting with primary outcomes and process outcomes (use).

	1	2	3	4	5	
subitem not at all important	○	○	○	○	◉	essential

Does your paper address subitem 22-i? *

Copy and paste relevant sections from the manuscript (include quotes in quotation marks "like this" to indicate direct quotes from your manuscript), or elaborate on this item by providing additional information not in the ms, or briefly explain why the item is not applicable/relevant for your study

"In this RCT, we found no significant difference in BMI change between the two groups at 6-months post-baseline. There were no significant differences in physical activity, screen-time or sleep outcomes between groups. The intervention did, however, demonstrate some positive outcomes in relation to dietary intake, child feeding and nutrition parent self-efficacy. To the best of our knowledge, Time2bHealthy is the first RCT to assess the efficacy of a parent-focussed healthy lifestyle intervention on BMI in preschool-aged children which is delivered entirely online".

22-ii) Highlight unanswered new questions, suggest future research

Highlight unanswered new questions, suggest future research.

	1	2	3	4	5	
subitem not at all important	○	○	○	○	◉	essential

Does your paper address subitem 22-ii?

Copy and paste relevant sections from the manuscript (include quotes in quotation marks "like this" to indicate direct quotes from your manuscript), or elaborate on this item by providing additional information not in the ms, or briefly explain why the item is not applicable/relevant for your study

"A cost-effectiveness analysis was not within the scope of this study. While it is generally perceived that that eHealth interventions are more cost-effective than traditionally-delivered programs, more research is needed [63]. Studies with a longer follow-up period and those which translate programs into primary health care are required to demonstrate long-term effectiveness".
"Future studies with a larger sample size, longer follow-up period and those that translate effective eHealth childhood obesity prevention programs into primary health care are needed".

20) Trial limitations, addressing sources of potential bias, imprecision, and, if relevant, multiplicity of analyses

20-i) Typical limitations in ehealth trials

Typical limitations in ehealth trials: Participants in ehealth trials are rarely blinded. Ehealth trials often look at a multiplicity of outcomes, increasing risk for a Type I error. Discuss biases due to non-use of the intervention/usability issues, biases through informed consent procedures, unexpected events.

	1	2	3	4	5	
subitem not at all important	○	○	○	○	◉	essential

Does your paper address subitem 20-i? *

Copy and paste relevant sections from the manuscript (include quotes in quotation marks "like this" to indicate direct quotes from your manuscript), or elaborate on this item by providing additional information not in the ms, or briefly explain why the item is not applicable/relevant for your study

"While it was intentional to include children of a healthy weight in this study, there were a higher than anticipated proportion of children (over 90%) in the healthy weight range. Therefore, the effect on BMI may have been diluted".
"As there were multiple outcomes assessed, there is a risk that there may have been a Type 1 error".
"Statistical power would have been affected by the fact that the target sample size was not achieved despite measures to enhance participant recruitment, including expanding the recruitment area and extending the recruitment period. It is also possible that a longer follow-up period may have been required to demonstrate differences in BMI change between groups".

21) Generalisability (external validity, applicability) of the trial findings

NPT: External validity of the trial findings according to the intervention, comparators, patients, and care providers or centers involved in the trial

21-i) Generalizability to other populations

Generalizability to other populations: In particular, discuss generalizability to a general Internet population, outside of a RCT setting, and general patient population, including applicability of the study results for other organizations

	1	2	3	4	5	
subitem not at all important	○	○	○	○	◉	essential

Does your paper address subitem 21-i?

Copy and paste relevant sections from the manuscript (include quotes in quotation marks "like this" to indicate direct quotes from your manuscript), or elaborate on this item by providing additional information not in the ms, or briefly explain why the item is not applicable/relevant for your study

"The weight status range of children in this sample demonstrates that the intervention can be applied to both healthy weight and overweight/obese children. However, further work is required to explore optimal avenues to access at-risk and hard to reach populations. It has been reported that the majority of parents do not recognise that their child is overweight and obese children [64]. Education and monitoring initiatives may therefore be useful to enhance parent awareness".

21-ii) Discuss if there were elements in the RCT that would be different in a routine application setting

Discuss if there were elements in the RCT that would be different in a routine application setting (e.g., prompts/reminders, more human involvement, training sessions or other co-**interventions**) and what impact the omission of these elements could have on use, adoption, or outcomes if the **intervention** is applied outside of a RCT setting.

	1	2	3	4	5	
subitem not at all important	○	○	○	○	◉	essential

Does your paper address subitem 21-ii?

Copy and paste relevant sections from the manuscript (include quotes in quotation marks "like this" to indicate direct quotes from your manuscript), or elaborate on this item by providing additional information not in the ms, or briefly explain why the item is not applicable/relevant for your study

"As this intervention is solely internet-based, it could be easily translated to a real-world setting given that most developed countries have a high proportion of internet users. The program would also be easily transferable to other countries by adapting the content to comply with local guidelines. In a real-world setting, data could be collected online which could improve participant retention, but lack of objectively measured data may create bias issues. It is recommended that further studies with a longer follow-up period and those which translate programs into primary health care be conducted to demonstrate long-term effectiveness".

OTHER INFORMATION

23) Registration number and name of trial registry

Does your paper address CONSORT subitem 23? *

Copy and paste relevant sections from the manuscript (include quotes in quotation marks "like this" to indicate direct quotes from your manuscript), or elaborate on this item by providing additional information not in the ms, or briefly explain why the item is not applicable/relevant for your study

"Trial Registration
Australian and New Zealand Clinical Trials Registry (12616000119493).
http://www.anzctr.org.au/"

24) Where the full trial protocol can be accessed, if available

Does your paper address CONSORT subitem 24? *

Cite a Multimedia Appendix, other reference, or copy and paste relevant sections from the manuscript (include quotes in quotation marks "like this" to indicate direct quotes from your manuscript), or elaborate on this item by providing additional information not in the ms, or briefly explain why the item is not applicable/relevant for your study

"The protocol for this study has been published [20]". Hammersley ML, Jones RA, and Okely AD, Time2bHealthy - An online childhood obesity prevention program for preschool-aged children: A randomised controlled trial protocol. Contemp Clin Trials 2017; 61:73-80.

25) Sources of funding and other support (such as supply of drugs), role of funders

Does your paper address CONSORT subitem 25? *

Copy and paste relevant sections from the manuscript (include quotes in quotation marks "like this" to indicate direct quotes from your manuscript), or elaborate on this item by providing additional information not in the ms, or briefly explain why the item is not applicable/relevant for your study

"This research has been conducted with the support of the Australian Government Research Training Program Scholarship. The study was also supported by funding from Australian Health Management. The funding body was not involved in the design, data collection, analysis, interpretation or writing".

X27) Conflicts of Interest (not a CONSORT item)

X27-i) State the relation of the study team towards the system being evaluated

In addition to the usual declaration of interests (financial or otherwise), also state the relation of the study team towards the system being evaluated, i.e., state if the authors/evaluators are distinct from or identical with the developers/sponsors of the intervention.

	1	2	3	4	5	
subitem not at all important	○	○	○	○	◉	essential

Does your paper address subitem X27-i?

Copy and paste relevant sections from the manuscript (include quotes in quotation marks "like this" to indicate direct quotes from your manuscript), or elaborate on this item by providing additional information not in the ms, or briefly explain why the item is not applicable/relevant for your study

"The University of Wollongong developed the Time2bHealthy program".

About the CONSORT EHEALTH checklist

As a result of using this checklist, did you make changes in your manuscript? *

- ○ yes, major changes
- ◉ yes, minor changes
- ○ no

What were the most important changes you made as a result of using this checklist?

Generalisability, number of participants completing each module.

How much time did you spend on going through the checklist INCLUDING making changes in your manuscript *

5 hours

As a result of using this checklist, do you think your manuscript has improved? *

- (●) yes
- ( ) no
- ( ) Other:

Would you like to become involved in the CONSORT EHEALTH group?

This would involve for example becoming involved in participating in a workshop and writing an "Explanation and Elaboration" document

- (●) yes
- ( ) no
- ( ) Other:

Any other comments or questions on CONSORT EHEALTH

Your answer

STOP - Save this form as PDF before you click submit

To generate a record that you filled in this form, we recommend to generate a PDF of this page (on a Mac, simply select "print" and then select "print as PDF") before you submit it.

When you submit your (revised) paper to JMIR, please upload the PDF as supplementary file.

Don't worry if some text in the textboxes is cut off, as we still have the complete information in our database. Thank you!

Final step: Click submit !

Click submit so we have your answers in our database!

SUBMIT

Never submit passwords through Google Forms.

This content is neither created nor endorsed by Google. Report Abuse - Terms of Service - Additional Terms

Google Forms
